# Variation isn’t that hard: Morphosyntactic choice does not predict production difficulty

**DOI:** 10.1371/journal.pone.0252602

**Published:** 2021-06-21

**Authors:** Matt Hunt Gardner, Eva Uffing, Nicholas Van Vaeck, Benedikt Szmrecsanyi

**Affiliations:** Department of Linguistics, KU Leuven, Leuven, Belgium; University of California Santa Barbara, UNITED STATES

## Abstract

The following paper explores the link between production difficulty and grammatical variability. Using a sub-sample of the Switchboard Corpus of American English (285 transcripts, 34 speakers), this paper shows that the presence of variable contexts does not positively correlate with two metrics of production difficulty, namely filled pauses (*um* and *uh*) and unfilled pauses (speech planning time). When 20 morphosyntactic variables are considered collectively (*N=* 6,268), there is no positive effect. In other words, variable contexts do not correlate with measurable production difficulties. These results challenge the view that grammatical variability is somehow sub-optimal for speakers, with additional burdensome cognitive planning.

## Introduction

This paper introduces a new research agenda aimed at exploring the link between language complexity and variation as inherent properties of language use. Specifically, we assess the complexity incurred by having to choose between competing grammatical variants.

As we will argue, while the existence of grammatical variation is often denied or deplored as a matter of doctrine, the assumption that grammatical variation creates complexities is not entirely unreasonable. The idea that grammatical variation might burden language production deserves scrutiny not primarily because language users are forced to make grammatical choices—after all, using language *always* entails plenty of choice-making—but rather, because grammatical variation (as opposed to e.g., lexical variation) is conditioned probabilistically by any number of contextual constraints. For example, Bresnan *et al.* [1] suggest that the English dative alternation (*give Anna an award* vs. *give an award to Anna*) is conditioned by as many as 10 probabilistic constraints, including e.g. constituent weight, constituent pronominality, information status, priming/persistence, and so on. Further, even before language users can make a choice as a function of the naturalness of grammatical variants in a specific linguistic context, they need to check that linguistic context for the various constraints that regulate the variation at hand (e.g. How long are the constituents? Are they pronominal? etc.). Regardless whether this analysis is automatic or under overt executive control (it is likely both to varying degrees as language users are demonstrably sensitive to probabilistic constraints [2]), the contextual analysis must happen at some level during language production. We ask the question, does this extra cognitive work unduly increase production difficulty?

An answer, one way or the other, has never been demonstrated empirically. Finding a strong positive correlation between indicators of increased cognitive load and grammatical variation would provide empirical evidence for the intuition that variation must be difficult and complex from a production standpoint, and is thus a sub-optimal facet of human communication tolerated given the separate social function of variation. Finding independence between these indicators and variation would challenge this intuition, and suggest that variation is not difficult, and does not inordinately burden language production. Finally, finding a strong negative correlation would point to facilitation, and suggest that variation might allow language users to actually optimize production, e.g., by signalling syntactic structure overtly, by maximizing communicative efficiency, by advantageously spreading information density, by satisfying universal tendencies, by aligning constituent order with information status, or by enabling desired prosodic patterns.

In order to explore the relationship between production difficulty and variation we employ the well-studied Switchboard Corpus of American English [3], looking for correlations between metrics of increased cognitive load and 20 different morphosyntactic variables. Below we report on an analysis of a subset of the corpus.

## Variation is hard‽

Our point of departure is the widespread prescriptivist gut feeling that variation is messy, and should therefore be eliminated (e.g., [4, 131–133, 686]). Uniformity of form and usage, in contrast, are to be desired. Many theoretical and descriptive linguists often treat variation as an exception to the norm (e.g., [5], for a review see [6, 7]). This idea—which critics have called the ‘Doctrine of Form-Function Symmetry’ [8]—fuels, for example, the ‘Principle of No Synonymy’ in cognitive linguistics [9, 67], or the principle of ‘isomorphism’ [10, 516]: if two grammatical forms exist, they must or should have a different meaning. Variation (i.e., the absence of meaning differentiation) is thus unpredicted and in need of (functional) explanation, and, by implication, should therefore be complex/difficult (or at least diachronically short-lived). But then again, we know that variation in language is universal and ubiquitous. Van Hout & Muysken [11] review how linguistics has come to terms with this chaos of language variation, namely, by either largely ignoring it (e.g. within the generative grammar framework) or integrating it in linguistic theory (e.g., in variationist sociolinguistics). Crucially, however, there is a dearth of empirical research investigating if variation is actually complex or difficult for language users in the first place.

The present paper addresses this gap by exploring the intersection between variationist linguistics and theorizing about language complexity. The variationist approach builds on the assumption that language is intrinsically and perpetually variable, though linguistic choices can be modeled by language-external as well as language-internal factors [12]. Language complexity, on the other hand, can either be of an absolute or a relative nature [13]. Absolute complexity focuses on the complexity of system-inherent structures and is assessed, e.g., by counting the number of contrastive elements in a system [14], by counting the amount of rules in a grammar [15], or by establishing the length of the shortest possible description of language samples [16]. These measures are built on system-level notions of complexity, i.e., on complexity inherent in an idealized, abstract system, as represented in reference grammars.

In contrast, relative complexity—which is the complexity notion that informs the approach in the present study—is defined as being proportional to cost and/or difficulty to language users [13]. Relative complexity measures thus often coincide with processing-related concepts and are measured, e.g., in terms of the iconicity of structures [17], in terms of communicative efficiency [18], or in terms of processing difficulty [19]. For our purposes, complexity is defined in relative terms and is specific to production: variation counts as complex if it makes production difficult for speakers.

We operationalize production difficulty in two ways: filled and unfilled pauses. Filled pauses (*um* and *uh* in North American English, as in (1)) are conventional, semantically-null sounds that occur in spontaneous speech that may represent intended or unintended hesitation by the speaker. Unfilled pauses (or speech planning time) is silence in the speech stream that occurs between words or utterances. We use filled and unfilled pauses as metrics for production difficulty: utterances with fewer filled pauses and/or shorter unfilled pauses are considered to have been easier to produce; utterances with more filled pauses or longer unfilled pauses are considered to have been harder to produce. This aligns with a stream of research that uses these metrics as indicators of a speaker’s cognitive load during production (as summarized by [20, 21]).

(1)
  a. **Uh** my husband and I just bought **uh** just moved here recently and **uh** we bought **uh** this house about, **uh** I don’t know, year and a half ago.
  (Female, South Midwest, born 1961)
  b. **Uh** it seems like, **uh** you know, the weather pattern is just shifting and **uh** that **uh** the jet stream is moving **uh** into our area of North Carolina
  (Female, South Midwest, born 1963)

Spontaneous speech takes place in real time, which means that speakers have to continually plan utterances both prior to and while speaking [22]. Unsurprisingly, this is a demanding task. As a consequence, disfluencies are non-trivial in spontaneous speech; across studies about 6% of uttered words are disfluent [23, 24]. Goldman-Eisler [25] shows that almost half of people’s speaking time is made up of pausing and overt disfluencies like *um* and *uh*. The occurrence of overt disfluencies like filled pauses and/or prolonged planning time have been used as evidence of planning difficulty or increased cognitive load on behalf of the speaker [20, 21]. They have also been shown to occur more frequently in contexts that are independently judged to be more difficult, for example, when utterances are longer or more syntactically complex [26–33], when the topic of conversation is unfamiliar [34, 35], when the discursive task is more challenging [36–39], or when lexical items are low frequency and/or have low contextual probability [40]. For example, Beattie & Butterworth [41] suggest that speakers are more aware when choosing unlikely words in a given context, and this choice may lead to increased disfluencies.

The notion that disfluency is linked to choice in particular has been present in studies on disfluencies since Goldman-Eisler [42, 97], who argued that speakers pause when they encounter uncertainty, or rather “when the selection of the next step requires an act of choice”. More recently Schnadt & Corley [43] found through a network test that an increase in possible lexical paths correlated with an increase in disfluencies, which they attributed to the additional processing load associated with speech planning introduced by lexical choice. Hartsuiker & Notebaert [44] additionally found that lexical items with low name agreement, i.e., words with multiple names, none of which are necessarily the first choice, tend to co-occur with disfluencies more frequently than lexical items with high name agreement. Conversely, Christenfeld [45] puts forward that in addition to lexical choice, “lexical suppression”, i.e., a lack of lexical options, may cause increased disfluency as well. An important note here is that all of these studies examine choice as it pertains to the lexicon.

Myachykov *et al.* [46] find in controlled experiments that Russian speakers have longer sentence onset latencies and eye-voice spans for subject-initial subject nouns (two markers of increased cognitive load while sentence planning) compared to English speakers when describing the action in a picture. The authors attribute the difference to the greater amount of competition from available syntactic alternatives in Russian compared to English and conclude that syntactic flexibility (i.e., grammatical variation) is cognitively costly. The study, however, did not compare makers of cognitive load when flexibility itself was controlled within each language—so whether the topological difference is reflected in a correlation between grammatical variation and increased cognitive load within each language is an open question.

**Fig 1:**
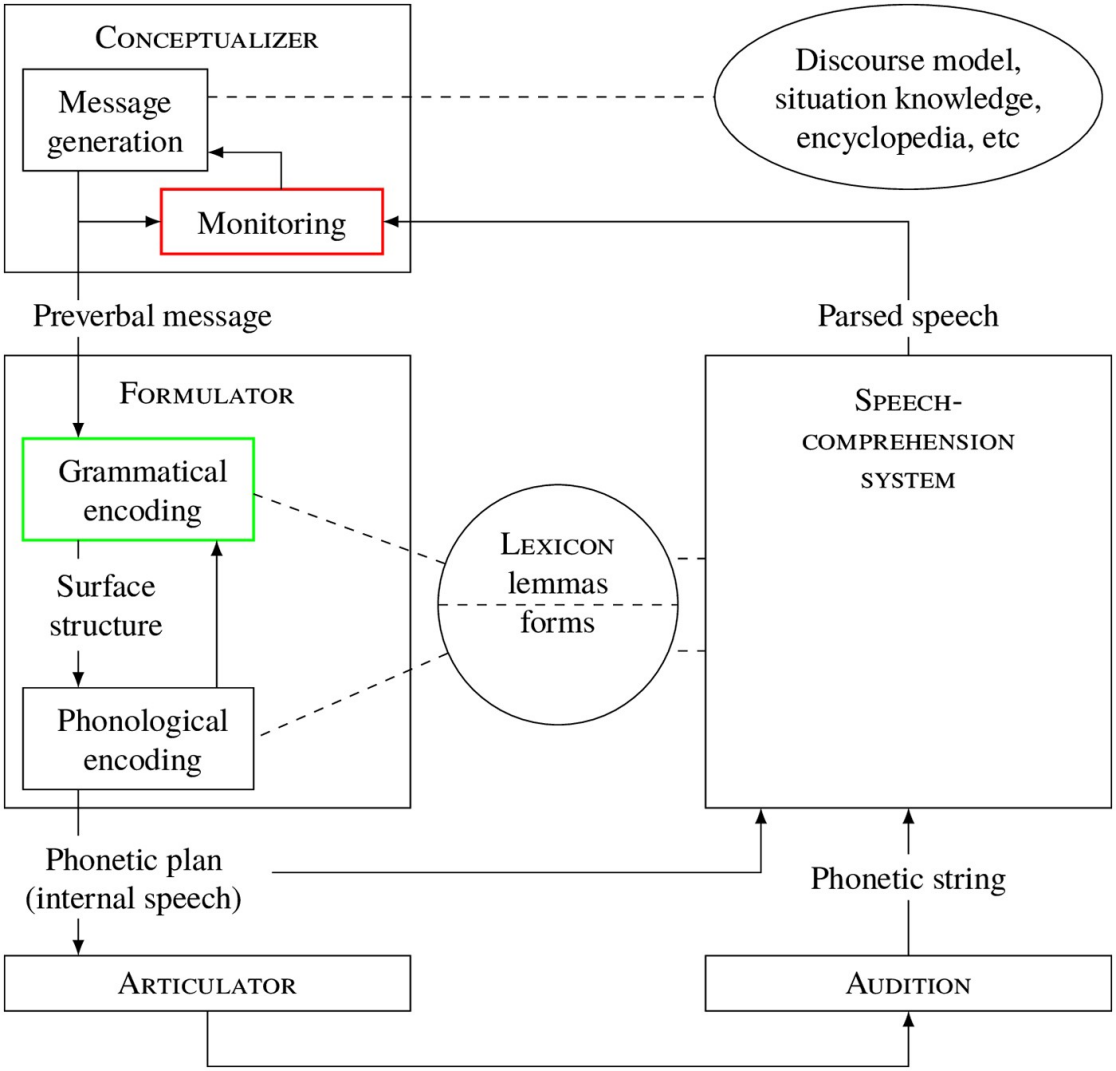
Levelt’s [49] model of speech production.

It is instructive to consider these findings in light of Garrod & Pickering’s [47] model of gradient automaticity (building on Baugh’s [48] four horseman of automaticity and Levelt’s [49, 50] model of speech production, reproduced as Fig 1). They state that speaking is a complex activity consisting of both automatic and controlled processes. The authors argue that “most aspects of language production involve some degree of choice between alternatives [i.e., control]. It may be that the degree of automaticity is related to the extent to which the speaker has to make such choices because choice relates to intentionality and strength of processing,” [47, 4].

Levelt’s [49] three broad stages of production are conceptualization (deciding what to talk about), formulation (constructing linguistic representations), and articulation (physically producing those representations). Garrod & Pickering [47] put forth that these three stages involve different degrees of automaticity and explicit executive control. Conceptualization exhibits high levels of control (especially during difficult discursive tasks). Formulation involves both morphosyntactic and phonological encoding—both involving less control and more automaticity—as well as an interface with lexical items encoded as lemmas, which informs both morphosyntactic and phonological encoding, and which involves more control than either. Finally articulation is the most automatic, though still involves some control (especially over total utterance prosodic features like tone of voice). If choice is linked to production difficulty, that difficulty ought to be most present in the conceptualization and lexical encoding steps because these steps involve the most explicit executive control, i.e., choice-making. Further, Garrod & Pickering [51] argue that there is a self-monitoring process during which salient variables may undergo variant substitution (though other models [49, 52] restrict this monitoring to phonetic or phonological monitoring, see also [53]). This relates to Labov’s [54] axiom that style ranges along a single dimension, measured by the amount of attention paid to speech. The more aware a speaker is of her speech, the more she adopts formal or prestigious speech features. The vernacular emerges in unmonitored speech. To synthesize, variable grammatical patterns may result from automatic processing (in unmonitored speech) or explicit choices (in monitored speech). The corollary of this is the hypothesis that there will be greater processing difficulty for highly-salient sociolinguistic variables (e.g., innovative *be like*) or grammatical alternations with overt style prescriptions (e.g., case and linear order of coordinated pronouns, which is subject to hypercorrection [55]).

An alternative view to Labov’s ‘attention-paid-to-speech’ axiom, is Bell’s notion of ‘audience design’, whereby style is “essentially speakers’ response to their audience” [56, 145]. Both overt disfluencies and speech planning time have been found to vary depending on who a speaker is speaking to. For example, Le Grézause [37] reports that rate of *um* vs. *uh* differ depending on dyad gender composition. Oviatt [28] documents that disfluency rates in human-computer interactions are substantially lower than rates typically observed during comparable human-human speech. Horton & Keyser’s [57] experimental results lead the authors to conclude that speakers do not engage in audience design in the initial planning of utterances; instead, they monitor planned utterances for violations related to shared knowledge, i.e., ‘common ground.’ This motivates the prediction that variation conditioned by audience design, and thus connected to a self-monitoring process, will likely coincide with more disfluency.

To summarize, there is overwhelming evidence that increased cognitive load coincides with increased rates of overt disfluencies (including filled pauses) and extended speech planning time (unfilled pauses). These two measures are in turn used as metrics for cognitive load. Exerting explicit executive control during production increases cognitive load, therefore choice-making is linked to disfluency and speech planning time. Grammatical planning involves both automatic processes and explicit control, and both must include probabilistic constraint-based variant selection. Variant substitution triggered by attention paid to speech or audience accommodation during the self-monitoring process is due to explicit executive control, adding to cognitive load, and potentially precipitating overt disfluencies or extended planning time. This leads to the hypothesis that grammatical alternations that are subject to style-shifting or audience design (i.e., sociolinguistic markers [58]) will coincide with a higher frequency of overt disfluencies and require more planning time compared to grammatical alternations that are not subject to style-shifting or audience design (i.e., sociolinguistic indicators [58]). Further, the greater the number of variable contexts subject to executive control within an utterance, the greater the number of overt disfluencies or the longer the amount of required planning time is expected

No research has been conducted thus far to investigate whether specifically *grammatical* choice, i.e., the presence of grammatical variation, may cause increased disfluency in spontaneous speech. As Abramovici [59, 101] notes, “in order to devise psychologically real grammars, one has to consider evidence that some linguists wish to exclude, such as socially determined linguistic variation and various performance errors—hesitations, false starts, slips of the tongue, and so forth—in addition to the evidence that linguists have usually considered”. This is precisely what we aim to do in the present study.

## Methods

### Data

For this paper we employ the Switchboard Corpus of American English [3]. This corpus consists of 2,438 spontaneous telephone conversations between 542 American English speakers recorded by Texas Instruments in 1989/1990. Conversations range from 5–10 min, and the full corpus totals 240 hours of recorded speech. Participants range from 20–60 years old and are categorized by dialect region, sex, and education level (see Table 1). All participants were ostensibly native speakers of English. Time-aligned transcripts were produced as part of the original project (generating ∼3 million words of text). This corpus is well-used, especially within the domain of psychololinguistics (we count over 400 citations), and has shown to be a useful tool for exploring linguistic phenomena.

**Table 1:** Summary of speaker demographics in the Switchboard corpus ([3], adapted from [60, 414].

Dialect	Age	Sex	Education
South Midland (155)	20–29 (140)	Male (229)	<High School (14)
Western (85)	30–39 (179)	Female (239)	High School, <College (39)
North Midland (77)	44–49 (112)		College (309)
Northern (75)	50–59 (87)		>College (176)
Southern (56)	60–69 (13)		Unknown (4)
New York City (33)			
Mixed (26)			
New England (21)			

The Switchboard Corpus has already been analyzed for overt disfluencies [26, 37, 61–64], though not in relation to grammatical variation. Le Grézause [37] reports a total of 10,784 *um*’s and 3,0187 *uh*’s across the full corpus, equaling 0.79% and 2.07% of total words respectively. Shriberg [26] reports a positive relationship between all overt disfluencies (filled pauses and others like restarts and repairs) and utterance length: longer utterances have more disfluencies. This pattern is found over the aggregate and for individual speakers. She also reports that filled pauses (*um* and *uh*) are more frequent than other types of overt disfluencies.

Below we present a new analysis focusing solely on young women (born in or after 1960) from the South Midland dialect (285 transcripts, 34 speakers). We do this to control for the known sex, age, and regional patterns reported by Wieling *et al.* [64]. The choice of young South Midland females was arbitrary, though this group is one of the largest demographic categories in the full corpus, giving us ample data in our sample. Further, young female speakers are predicted to employ fewer non-standard or region-specific variants [65]. As region-specific patterns are explicitly not in the purview of this study, we consider this an advantage. The Switchboard Corpus does not provide additional demographic information about its speakers; therefore, additional known factors that correlate with variable patterns, like ethnicity or multilingualism, cannot be operationalized or controlled.

Not all conversations involving South Midland young females were previously parsed for disfluent phenomena, so additional by-line coding of the data for disfluent phenomena following the original parsing protocol [66] was required.

**Fig 2:**
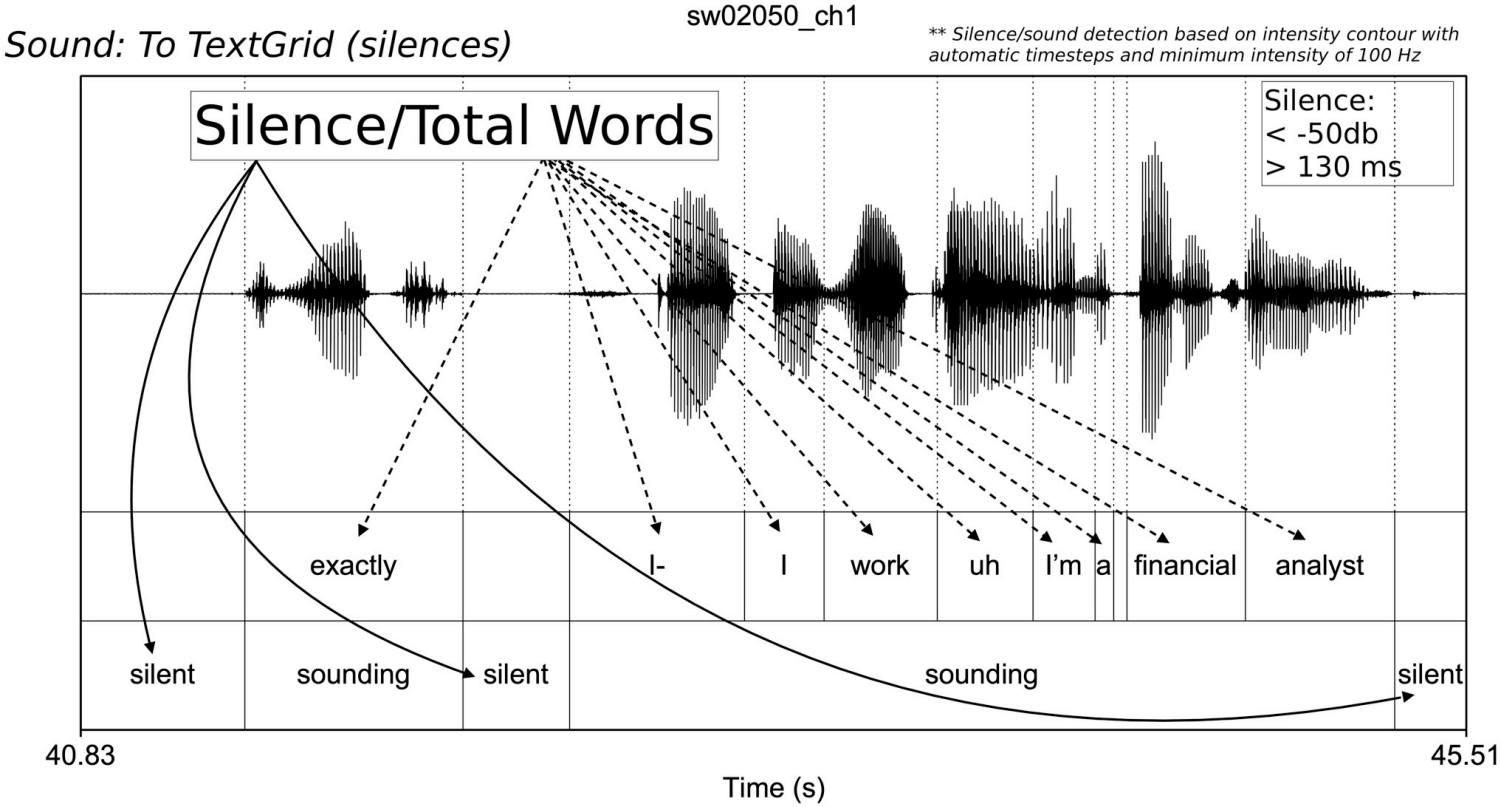
Example waveform and silence annotation using *Praat* [67]. Mean silence per word calculated by dividing total silence time (in ms) by total number of words.

For our analysis we take each speaker turn as an individual data point. Conversations occur between young South Midland speakers and speakers from across the United States; however, we only consider the speech of the young South Midland female participants. A turn is defined as speech (and its accompanying silence) by a speaker that occurs between the utterances of her interlocutor. We also only include turns longer than three words as many of the variable contexts examined below cannot occur in such short utterances. This also eliminates backchannels. A total of 7,161 turns are included in our analysis. For each turn we count the number of filled pauses and the number of canonical words. The length of unfilled pauses, or speech planning time, was determined using the built-in silence detection script (*Sound: To TextGrid (silences)*) in *Praat* [67] (see Fig 2), which detects sounding and silent intervals based on the intensity of the audio stream. Silence was defined as a portion of the audio stream below -50 dB and longer than 130 ms (following [68]). While speech planning undoubtedly occurs concurrent with listening, for practical purposes, we restrict our definition to speech planning time that is turn-internal. We record the total amount of silence per turn (in seconds); we also divide the total amount of silence by the number of words uttered during a turn to achieve a value for mean silence per word. This normalizing by word offsets differences in total amount of silence per turn across turns of varying lengths.

## Grammatical variation

At the heart of all variationist research is the linguistic variable, which can be expressed by two or more variants that are semantically (near)-equivalent and therefore interchangeable because they constitute “alternative ways of saying ‘the same thing”’ [58, 88]. In the realm of grammatical variation, variables are also known as ‘alternations’. This paper is specifically interested in variation between grammatical variants that are, in principle, available to all members of the speech community, regardless of dialect background (i.e., Type 3 variables following [11]).

We sought to compile a list of as many major variables in mainstream North American English as possible. The 20 variables happen to represent a broad spectrum of types of variation. Some variables are considered stable diachronically, others represent changes in progress occurring slowly over hundreds of years, while quotatives are a rapid change in progress. Some variables may have no stylistic or register associations, while others may have one or more variants that are prescribed and formal, or alternatively that are common in informal speech.

We were also careful *not* to select variables for which (nearly) every sentence would represent a variable context, e.g., active vs. passive voice.

It is beyond the scope of this paper to provide a full review of the pertinent literature describing each of these variables; however, our inclusion and exclusion criteria are carefully considered and match current practice where possible. Across variables we exclude all instances in which the variable context occurs as part of a fixed expression or invariable proper name, for example: (2a) for the genitive alternation; (2b) for the dative alternation; and (2c) for expressions of future temporal reference.

(2)
  a.
    Yeah there’s uh– **Biraporetti’s** is one of our favourites, it’s real nice, it’s Italian.
    (Female, South Midland, born 1952)
  b
    We didn’t no more need no new engine. **Give me a break**, you know.
    (Female, South Midland, born 1963)
  c
    One was Jewish and one was Black and this all took place in the South and uh normally **never the twain shall meet**.
    (Female, South Midland, born 1956)

We list the 20 variables below.

### Pronouns

#### Indefinite pronouns with singular human reference

The set of English human pronominal quantifiers occur with equal semantic meaning as compound forms with *-one* or *-body* in Modern English [69, 70]. These include *someone/somebody*, *anyone/anybody*, and *no one/nobody*.

(3)
  a
    **Everybody** has on like gloves and scarves and ear muffs and everything
    (Female, South Midland, born 1970)
  b
    You get to hear the whole scoop on **everyone** because **everyone** would know.
    (Female, South Midland, born 1970)

Of the 247 indefinite pronouns with singular human reference in the young South Midland female data, 163 (66%) are -*body* forms and 84 (34%) are -*one* forms.

#### Coordinated pronouns

There is variation in both the case and linear order of coordinated first- and third-person pronouns in either subject or object position [55, 71–74]. The pronouns may be coordinated with any NP. Case variation for second person pronouns is opaque, as in (4f), but linear order is not, therefore linear order is considered for second-person pronouns where they occur in coordinated structures.

(4)
  a
    It’s a topic that, um, **my husband and I** often discuss
    (Female, South Midland, born 1961)
  b
    Well, **me and my husband** are. We’ve talked about it.
    (Female, South Midland, born 1965)
  c
    It’s sort of a bone of contention between **she and my dad**.
    (Female, South Midland, born 1961)
  d
    I work for a really small company; it’s just **me and my boss**.
    (Female, South Midland, born 1970)
  e
    I got the the Airedale for my husband for a Christmas present and the Peekaboo was mine for **my girls and me**.
    (Female, South Midland, born 1963)
  f
    When they declare bankruptcy it’s **you and me** that- that have to pay for it.
    (Female, South Midland, born 1963)

There are 40 instances of coordinated pronouns in the young female South Midland data. In the 29 tokens in which the coordinated pronouns are subjects, 23 (79%) are prescriptively correct nominative forms. In the 11 tokens in which the coordinated pronouns are objects, 9 (82%) are the prescriptively correct accusative/dative forms. For coordinated subjects, the pronoun is preceded by the NP in 20 tokens (69%) and follows by the NP in 8 tokens (28%). In 1 token two pronouns are coordinated (*she and I*). For coordinated objects, the pronoun is preceded by the NP in 6 tokens (55%) and followed by the NP in 2 tokens (18%). In 4 tokens two pronouns are coordinated (*you and me*; *us and them*, etc.).

### Complements

#### *That* versus zero complementizers

In English, speakers sometimes omit the complementizer *that* [75–78]. This alternation occurs primarily after matrix verbs *think*, *say*, and *know*, though it does occur elsewhere (see [79, 80]).

(5)
    I don’t think_____it’s a deterrent at all. I don’t think **that** anyone that, uh, commits a murder actually thinks **that** they will ever even uh be punished let alone put to death for it.
    (Female, South Midland, born 1961)

In 864 (83%) of the 1,041 tokens of this variable among young South Midland females complementizer *that* is omitted.

#### Infinitival versus gerundial complementation

After some head verbs there is a choice between two types of non-finite verbal complements: infinitival complementation, as in (6a), or gerundial complementation, as in (6b) [81–84]. There are four classes of verbs after which this variation regularly occurs. Following Szmrecsanyi [85], we focus on two: emotive verbs (*dread, hate, like, loathe, love, prefer*, etc.); and aspectual verbs of beginning, continuing, and ending (*start, begin, continue, cease*, etc.).

(6)
  a
    I love **to play** racquetball.
    (Female, South Midland, born 1972)
    I hate **mowing**.
    (Female, South Midland, born 1961)

There are 50 tokens of this variable among young South Midland females; 29 (58%) are infinitival complementation.

#### *Remember, regret, deny* complementation

After some verbs (*remember*, *regret*, *deny*) there is the choice between a *that*-clause and a gerundial (*-ing*) clause, as in (7) [83]. Where *that* can occur, *that* can also be omitted, as in (7c). There are 19 instances of this variable among young South Midland women, 11 (58%) instances are gerundial clauses, while the remaining were *that*-clauses (of which only 2 of 8 have overt *that*).

(7)
  a
    I don’t remember **being** that picky about my clothes.
    (Female, South Midland, born 1961)
  b
    I remember in Driver’s Ed in high school **that- that** my teacher always liked to listen to country music as we were driving.
    (Female, South Midland, 1961)
  c
    I remember______Chucky Cheeses was down in Lewisville.
    (Female, South Midland, born 1965)

#### *Try* complementation

After the verb *try*, various complementation strategies are possible [86–88]. In the present tense/imperative/infinitive, *try* can be followed variably by the usual subordinator *to*, *and* acting as a marker of infinitival subordination, or a gerund (as in 8). In the past tense only the subordinator *to* and a gerund are possible (as in 9), therefore present tense/imperative/infinitive *try* and past tense *try* represent two separate variables in our analysis.

(8)
  a
    I **try to** take them away from him.
    (Female, South Midland, born 1964)
  b
    They get you at the ticket booth and then **try and** get you at the popcorn and Coke.
    (Female, South Midland, born 1964)
  c
    So he wants to **try doing** some, you know, different kind of stuff.
    (Female, South Midland, born 1961)

(9)
  a
    When I **tried to** close with Chase Manhattan, it was just like pulling teeth.
    (Female, South Midland, born 1964)
  b
    We **tried using** Chem Lawn, but, um, our problem is our front yard is completely different from the backyard.
    (Female, South Midland, born 1961)

There are 47 present tense/imperative/infinitive tokens in the young South Midland female data; 43 (91%) are *try to*, 3 (7%) are *try and*, and one is *try* + gerund. There are 14 past tense tokens; all but one are *tried to*.

### Syntactic order

#### Particle placement

This variation is specific to transitive particle verbs (also called phrasal verbs), for which the placement of the particle can be either before or after the direct object [89–92].

(10)
    They tried really hard to **pay them off** and it took them three years before they were able to uh **pay off their credit cards**.
    (Female, South Midland, born 1970)

According to Lohse *et al.* [93] (see also [94]) there are 750 instances of this variation in the Switchboard Corpus. In 31% of the instances, the direct object precedes the particle, as in *pay them off* (10). However, among just the young South Midland females (*N* = 236) the direct object precedes the particle 73% of the time (*N* = 172).

#### Dative alternation

English users can switch the order of recipient and theme in ditransitive verb constructions [1, 95–99]. Szmrecsanyi *et al.* [100] (based on [101]) reports 1,221 instances of this variation in the Switchboard Corpus for just the verb *to give*. The “ditransitive dative”, as in *someone’s given me one* (11), occurred in 85.7% of the *to give* tokens, while the “prepositional dative”, as in *given it to me* (11), occurred in 14.3% of the *to give* tokens. Unlike Szmrecsanyi *et al.* [100] we consider all ditransitive verbs that take part in this alternation.

(11)
    I’ve never even bought a gun myself. My dad’s **given it to me** or **someone’s given me one**. So I’m probably real illegal, you know, carrying guns that aren’t even mine.
    (Female, South Midland, born 1967)

There are 39 ditransitive verb tokens from young South Midland females, of which 12 (31%) are the prepositional dative.

#### Genitive alternation

Possession by non-proform NPs can be expressed in two ways in English: using the “*s*-genitive” (12a) and the “*of*-genitive” (12b). Possession by pro-forms is expressed in two similar constructions: using a possessive determiner (12c) or an *of* + genitive pronoun (12d). Bare genitive pronouns (12) are also possible, though not included in our analysis. Our full inclusion-exclusion criteria follow Shih *et al.* [102] (see also [103–108]).

(12)
  a
    I mean they took someone else**’s** life so I don’t I just don’t think they should deserve to live
    (Female, South Midland, born 1970)
  b
    They also should respect the sanctity **of** the American home.
    (Male, South Midland, born 1964)
  c
    As soon as the killer kills someone they- they should deserve to die, **their** life is over too.
    (Female, South Midland, born 1970)
  d
    I had some friends **of mine** uh get sent up to Stanford for a year for uh college.
    (Female, South Midland, born 1970).
  e
    That’s what year **mine** is.
    (Male, South Midland, born 1961)

The data from Szmrecsanyi *et al.* [100] (based on [102]) shows 1,120 instances of NP genitive patterns in the Switchboard Corpus. Of these, 41% occurred with the *s*-genitive and 59% occurred with the *of*-genitive. Among young South Midland females there are 1,648 tokens of possession, of which 1,582 (96%) are pre-nominal possessive determiners. Of the remaining 66 tokens, 46 (70%) are *s*-genitives, and 20 (30%) are *of*-genitives.

### Relativizers

#### Restrictive relativizers

Restrictive relative clauses “serve to identify their antecedent” [109, 278], restricting or defining the meaning of a noun or noun phrase [104]. Restrictive relative clauses can be introduced by *that*, certain *wh*-forms, or zero [104, 110, 111], as in (13), though *that* is often the prescribed variant (leaving *which* for nonrestricted relative clauses) [4, 112].

(13)
  a
    We’ve uh- we’ve been married for about ten years and we find out that, you know, no matter what kind of budget_____you stick on, there’s always going to be an unexpected car repair or something happen with the house **that** you have to have money for.
    (Female, South Midland, born 1972)
  b
    If when they’re meeting with the engineers, **who** they know are going to be dressed down, if they come in, you know, in a six hundred dollar three piece suit, it’s gonna make the people they’re meeting with feel very uncomfortable.
    (Female, South Midland, born 1963)

There are 257 instances of restrictive relative clauses in the data from young South Midland female speakers. None are are introduced by a *wh*-form, 154 (60%) are introduced by *that*, and 103 (40%) have no overt relativizer.

#### Nonrestrictive relativizers

A nonrestrictive relative clause adds additional, descriptive information to a sentence [104]. Nonrestrictive relative clauses, as in 14, are semantically distinct from restrictive relative clauses and are always introduced by a *wh*-form or *that*, but never by zero in mainstream North American English.

Generally, unless prosodic clues are used, it is often difficult to discern whether a relative clause is restrictive or nonrestrictive [104]. As noted above, there is also historical prescription that nonrestrictive relative clauses are introduced by *wh-*forms [4], and so it is often the use of a *wh-*form that grammarians use to categorize a token as restrictive or nonrestrictive. Given tokens like (14), however, it is clear that variation between *that* and *wh-*forms does occur in nonrestrictive contexts.

To differentiate restrictive and nonrestrictive relative clauses we use the following diagnostic: if the relativizer can be felicitously replaced with zero (i.e., omitted) the clause is restrictive (as in 13), if not, it is nonrestrictive (as in 14). Additionally, restrictive relative clauses consist of a relativizer (including zero) followed by a subject and verb, nonrestrictive relative clauses consist of a relativizer (never zero) followed by a verb. This is admittedly a blunt strategy (and somewhat inconsistent with studies focused exclusively on relativizers, e.g.,[111, 113, 114]); however, as our goal is to identify variable contexts overall, whether a token is classified as a restrictive or nonrestrictive relative clause is moot, as the token still adds to the overall count of variable contexts. If there is a divergent effect vis-à-vis production difficulty between these clause categories, more fine-grained categorization may be required.

(14)
    Somebody like you, **that**’s used to having good quality sneaks [sneakers], would really hate to give up, um, good quality sneaks for a better price, but somebody like me, **who**’s not used to having good quality sneaks, would hate to pay more for better quality sneaks.
    (Male, South, born 1961)

In the data from young South Midland females, there are 206 tokens of nonrestrictive relative clauses; 147 tokens (71%) are introduced by *that*, 59 tokens (29%) are introduced by a *wh*-form.

### Morphology/non-lexical substitution

#### Analytic vs. synthetic comparatives

In English, comparative adjectives can be formed synthetically using the suffix *-er* (15a), or analytically with the addition of the adverb *more* (15b) [115, 116, 117].

(15)
  a
    I love going outside and stuff when it’s **warmer** and stuff.
    (Female, South Midland, born 1970)
  b
    I think in Dallas it’s a lot **more scary** just because it’s a big city.
    (Female, South Midland, born 1970)

Only comparative adjectives that vary in their use of either a synthetic or analytical form in the overall dataset are included in our analysis. Among the young Midlands female speakers there are only 32 tokens of these adjectives, of which 18 (56%) are synthetic comparatives.

#### *There is/was* with plural subjects

Non-standard *to be* agreement is a widespread feature of English varieties. Here, we focus on variation between singular *is/was* and plural *are/were* with plural subjects, as in (16). Though this variation may occur for all subject types, it is most common for plural existentials with *there* [118–120]; therefore, we restrict our analysis to plural existentials.

(16)
    Right because then **there’s** some places, and I would- and I would- don’t- this would be a- a big minus for the places, **there are** some places that state that you cannot come in unannounced. (Female, South Midland, born 1970)

In the data from the young female South Midland speakers there are 53 instances of plural existentials; 40 tokens (75%) occur with singular *is/was*.

#### Expressions of future temporal reference

The English tense system does not include a morphological future tense. Instead, it only distinguishes between past and non-past [103]. In order to refer to future time several strategies are possible, of which modal *will* and semi-modal *be going to* are the most common in Modern English, as in (17). Minor variants include *shall*, present progressive, *fixing to*, etc., [103, 121–124]. This variation is restricted to present tense morphology.

(17)
    I think eventually it **will** happen, you know, it**’s** just **going to** take- it**’s** just **going to** take a lot of education and a lot of time.
    (Female, South Midland, born 1963)

There are 630 tokens of future temporal reference in the data from young South Midland females; 232 tokens (38%) are *be going to*, 385 tokens (61%) are *will*, 1 token is *shall*, 2 are *fixing to*, and the remaining 10 are simple present/present progressive.

#### Expressions of deontic modality

In English, deontic modality is usually expressed using one of five forms: *must*, *have to*, *have got to*, *got to*, or *need to*, as in (18) [125, 126]. As with future temporal reference, this variation occurs only in the present tense. The first three variants can also express epistemic modality, as in (19); these variants with this function are not included in the present analysis.

(18)
  a
    I **must** admit I- I used to try to watch it.
    (Female, South Midland, born 1924)
  b
    When you see a a job that **needs to** be done, sometimes you **have to** fill out the five forms in triplicate in order to to get it done.
    (Female, South Midland, born 1970)
  c
    It’s like you **got to** find where you fit in best.
    (Female, South Midland, born 1970)
  d
    You**’ve got to** have good adult mentors.
    (Female, South Midland, born 1970)

(19)
    Yeah put the safety on the gun. Don’t assume- always assume it’s loaded that’s- that**’s got to** be the most common thing that people- yeah, it’s “Oh it’s empty. It’s empty.” “Boom!” You know.
    (Female, South Midland, born 1961)

Of the 330 deontic modality tokens from young South Midland females there are 228 tokens of *have to* (68%), 55 tokens of *need to* (17%), 19 tokens of *got to* (6%), 16 tokens of *have got to* (5%), and 12 tokens of *must* (4%).

#### Expression of stative possession

Stative possession is usually expressed using one of three forms: *have*, *have got*, or *got*, as in (20) [127–129]. Stative possession is also expressed, albeit less frequently, with verbs like *possess*, *hold* (a degree), *be endowed with*, etc. As with the two previous variables, this variation exists only in present tense contexts.

(20)
  a
    Yeah I**’ve got** the kits to to put them all in, I don’t **have** them all yet.
    (Female, South Midland, born 1963)
  b
    You sound like you **got** a Louisiana accent
    (Female, South Midland, 1965)

A total of 564 stative possessive tokens occur in the South Midland young female data. There are 321 tokens of *have* (57%), 214 tokens of *have got* (38%), and 29 tokens of *got* (5%).

#### Quotatives

The choice of verb for introducing a direct quotation has undergone rapid change in the second-half of the 20th century. Speakers categorized as ‘young’ in 1990, when the Switchboard Corpus was collected, are considered the first generation of robust users of the innovative *be like* form [130, 131], etc. Other verbs include *say, think, go, ask, tell*, and a collection of other semantically rich, yet infrequent, forms like *whisper, yell*, and *retort*.

Our extraction criteria are modelled after Tagliamonte & D’Arcy [132], thus all instances of verbs introducing ‘constructed dialogue’ [133], whether reported speech or reported internal thought, were extracted. Instances of direct quotation with no overt quotative verb, as in (21d), were also extracted.

(21)
  a
    You know, we get this lackadaisical attitude and **say** “Huh, you know, why should we vote?”
    (Female, South Midland, born 1963)
  b
    I **was like**, “No way!” and she **goes**, “Yeah!” She **goes**, “You know people get broken into a lot because they, you know, it’s real easy to get away with there, you know, no one can see them carrying out the stuff if no one lives nearby.”
    (Female, South Midland, born 1970)
  c
    The coach actually called time out and came and tapped my tail with her foot and said, you know pointed that finger, and **went**, “Ah, yeah, yeah, yeah, yeah!” you know, and I **thought**, “Okay.”
    (Female, South Midlands, born 1963)
  d
    I don’t know. I like that he walks in, you know, and he’s got this little gift and,_____“How’d you pay for it?”_____“Credit.” He, you know, **said**, “Whipped out my little card.”(Female, South Midlands, born 1965)

Verbs like *say* and *think* can also introduce indirect speech, usually followed by *that*. This *that* complementizer varies with a zero complementizer. We meticulously checked each instance of *say*/*think*, etc., to asses whether the token is an instance of a verb of direct quotation, as in (21), or a verb of indirect quotation with a zero complementizer, as in (22).

(22)
    I asked the vet why he did that and he said_____he was probably weaned too young, which he was, because his mom was killed, so, but he is the lovingest cat.
    (Female, South Midland, born 1967)

Buchstaller [134] previously examined quotatives in the Switchboard Corpus, finding that, of the 1,371 tokens across the full dataset, there were 121 tokens (9%) of innovative *be like* and 80 tokens (7%) of innovative *go* (see also [135]). Both forms were most frequent among those born after the mid-1960s. We find 120 instances of direct quotation among the young South Midland female data (all born after 1960), of which 34 (28%) are *be like*, 38 (32%) are *say*, 13 (11%) are *think*, 8 (7%) are *go*, 13 (11%) have no overt quotative, and the remaining 14 (12%) are some other quotative verb.

### Negation

#### *Not* versus *no* negation

In English there are three equivalent negative structures, as in Table 2 and (23), that involve *not* and *any-*.

**Table 2:** Forms within the variable context, based on Childs [136].

*Not* -negation	*No* -negation	Negative concord
*not…any*	*no, none*	*not…no/none*
*not…anybody*	*nobody*	*not…nobody*
*not…anyone*	*no one*	*not…no one*
*not…anything*	*nothing*	*not…nothing*
*not…anywhere*	*nowhere*	*not…nowhere*

(23)
  a
    There’s just **not any** push to recycle
    (Female, South Midland, born 1965)
  b
    There’s **nobody** to- to sit them down and say, “You’re going to do this.”
    (Female, South Midland, born 1963)
  c
    There’s **not nobody** can take care of your kids better than you (Female, South Midland, born 1964)

Excluded from this variation, based on Childs [136] (see also [137–139]), are constructions with only a constituent negation reading; with *a/an* instead of *any*; that are pre-verbal indefinites; that are general extenders; or with negated adjectives.

In the young South Midland female data there are 195 variable contexts: 149 are *not*-negation (76%), 44 are *no*-negation (23%), and the remaining 2 are negative concord (1%).

#### *Not* versus auxiliary contraction

In English the auxiliaries *had*, *has*, *have*, *will*, *would*, *is*, *are*, and *am* can be contracted on the right side of a subject NP. Likewise, *not*, can be contracted with a verb. In Late Modern English *not* contraction is exclusive to auxiliary and modal verbs. This gives rise to variation between auxiliary/modal contraction, as in *he’s* (24a), and *not* contraction, as in *isn’t* (24b) [140, 141].

(24)
  a
    I mean, he **wouldn’t** admit to you that he **doesn’t** like it, but it, you know, he**’s not** doing good and he never is excited about it or anything, and he **won’t** tell his parents.
    (Female, South Midlands, born 1970)
  b
    That**’s not** very fun- fun- fun- you know, that**’s not** very far to run and a month **isn’t** very long
    (Female, South Midlands, born 1972)
  c
    You know it’s supposed to be all for one and one for all, but that **ain’t** how it works, baby.
    (Female, South Midland, born 1963)

The form *am* cannot be contracted with *not* in mainstream North American English, so forms such as *I’m not* are outside the envelope of variation. The contracted first person form is, however, one possible origin of *ain’t* ([142], etc.). *Ain’t* is also a licit variant for other auxiliaries, including *do*, which does not participate in this variation. There are 28 instances of *ain’t* across the entire Switchboard Corpus. Given that none occur with first person singular *I*, all instances of *ain’t* in non-*do* contexts are included in the analysis as *not* contractions.

There are 500 contexts in the young South Midland female data where this variation can occur. Of these contexts, 201 (40%) are auxiliary contractions, and 299 (60%) are *not* contractions. Of the *not* contraction forms only 1 is *ain’t*.

## Questions and answers about our methodology

A number of critical questions may be raised concerning our methodology (and we are grateful to our reviewers for pointing them out):

*Why is the unit of analysis in our study chunks of text/speech (i.e. conversational turns), and not individual choice points?* It is important to emphasize that the dependent variables in our study are two measures of disfluency: filled and unfilled pauses. We predict these disfluencies based on, among other things, the number of grammatical variable contexts within the nearby linguistic environment. It is nearly impossible to define the precise syntactic or discursive points at which disfluencies *could* occur. The reason is that disfluencies can occur pretty much anytime, anywhere. We would therefore need to consider an infinite number of potential choice points, which is not operational. Further, as is well attested in the psycholinguistic literature (e.g., [143]), speech planning begins even before an interlocutor’s turn ends, so the choice-making required while planning a turn containing a variable context may occur over a much larger time domain than the exact temporal point at which an individual alternation occurs, and therefore a disfluency that is a reflex of that choice may also occur anywhere within that larger time domain.
*Why do we not control for syntactic priming? Isn’t priming relevant for determining how much choice and control the speaker actually has?* As customary in the corpus-based literature on priming [85, 144], we consider priming to be one probabilistic constraint on (and predictor for) variant choice among potentially many. Seen in this light, (a) it is not clear that priming reduces control any more than other constraints (e.g. animacy, end weight) do; (b) however mechanistic a constraint priming is, language users are still sensitive to competing probabilistic constraints (and have to check the linguistic context accordingly); and (c) it is conceivable that priming might make choices even harder, as when the presence of a prime favors choice of variant *x* but other probabilistic constraints favor choice of variant *y*. More generally, in this study we focus on the effect of the presence of variable contexts (i.e., choice points), without modeling these choices themselves (see also next question).
*What is the rationale for treating all variation contexts the same, regardless of the context-specific probability that a particular variant is chosen?* On practical grounds, we are working with aggregated data (see above), and operationalizing details of each individual variable context is non-trivial. There is no baseline measure of the “choicy-ness” associated with specific contexts in which our 20 variables occur. The probability of a variant of a variable for a specific speaker, in a specific turn, in this specific data, is an empirical question, not something known *a priori*. On more theoretical grounds, the assumption underlying this question is that variation contexts where one variant has a high probability of occurring are somehow less “choicy” than, say, variation contexts where all variants have roughly the same probability of occurring. This is certainly an interesting hypothesis that needs to be tested in separate research. However, we assert that regardless of what the relationship between probability and “choicy-ness” is, speakers must, in any event, first recognize variable contexts and then identify and weigh the constellation of linguistic (and, potentially, social) factors that influence (however strongly) the choice of variant in the given context. The same process necessarily occurs for all loci of variation, and presumably increases cognitive load—though this is exactly what we aim to test. In short, in the absence of empirical research addressing the issue of “choicy-ness” we prefer to err on the side of conservativeness by making fewer assumptions and treating all variation contexts the same.

## Results: Exploring the relationship between processing difficulty and variability

The first challenge in exploring the relationship between processing difficulty and variability involves deciding how to model the number of variable contexts in the data, as well as the two measures of disfluency. The distribution of each of these three variables—number of variable contexts, number of filled pauses, amount of silence—poses a problem for regression analysis.

Across our sub-sample of 285 audio files there were 7,161 total turns. Across the data, 6,268 variable contexts occur; however, they are restricted to just 3,468 (48%) turns (see Table 3). Further, the number of variable contexts per turn ranges in whole numbers from 1 through 11, though more than half of these are 1. Given the large number of turns with no variable contexts, and the zero-bound (i.e., no values <0), right-skewed (i.e., non-normal, long right tail) distribution of variable contexts per turn, we chose to consider variable contexts as a binary rather than as an ordinal variable in the analyses we report below. In other words, we classify turns as either containing at least one variable context (*N* = 3,468) or containing no variable contexts (*N* = 3,693).

**Table 3:** Distribution of number of variable contexts per turn among young South Midland speakers in the Switchboard Corpus.

Variable Contexts	Turns	%
0	3,693	52%
1	1,879	26%
2	897	13%
3	388	5%
4	181	3%
5	67	1%
6	39	<1%
7	8	<1%
8	3	<1%
9	3	<1%
10	2	<1%
11	1	<1%

Similarly, the distribution of filled pauses is zero-bound and heavily right-skewed. As Table 4 shows, of the 7,161 turns, 2,161 (30%) contain filled pauses, and the maximum number of filled pauses per turn is 5. For simplicity, in the analyses we report below, we treat number of filled pauses as a binary variable, with each turn either containing (*N* = 2,161) or not containing (*N* = 5,000) at least one filled pause.

**Table 4:** Distribution of number of filled pauses per turn among young South Midland speakers in the Switchboard Corpus.

Filled Pauses	Turns	%
0	5,000	70%
1	1,559	22%
2	461	6%
3	115	2%
4	22	<1%
5	4	<1%

**Fig 3:**
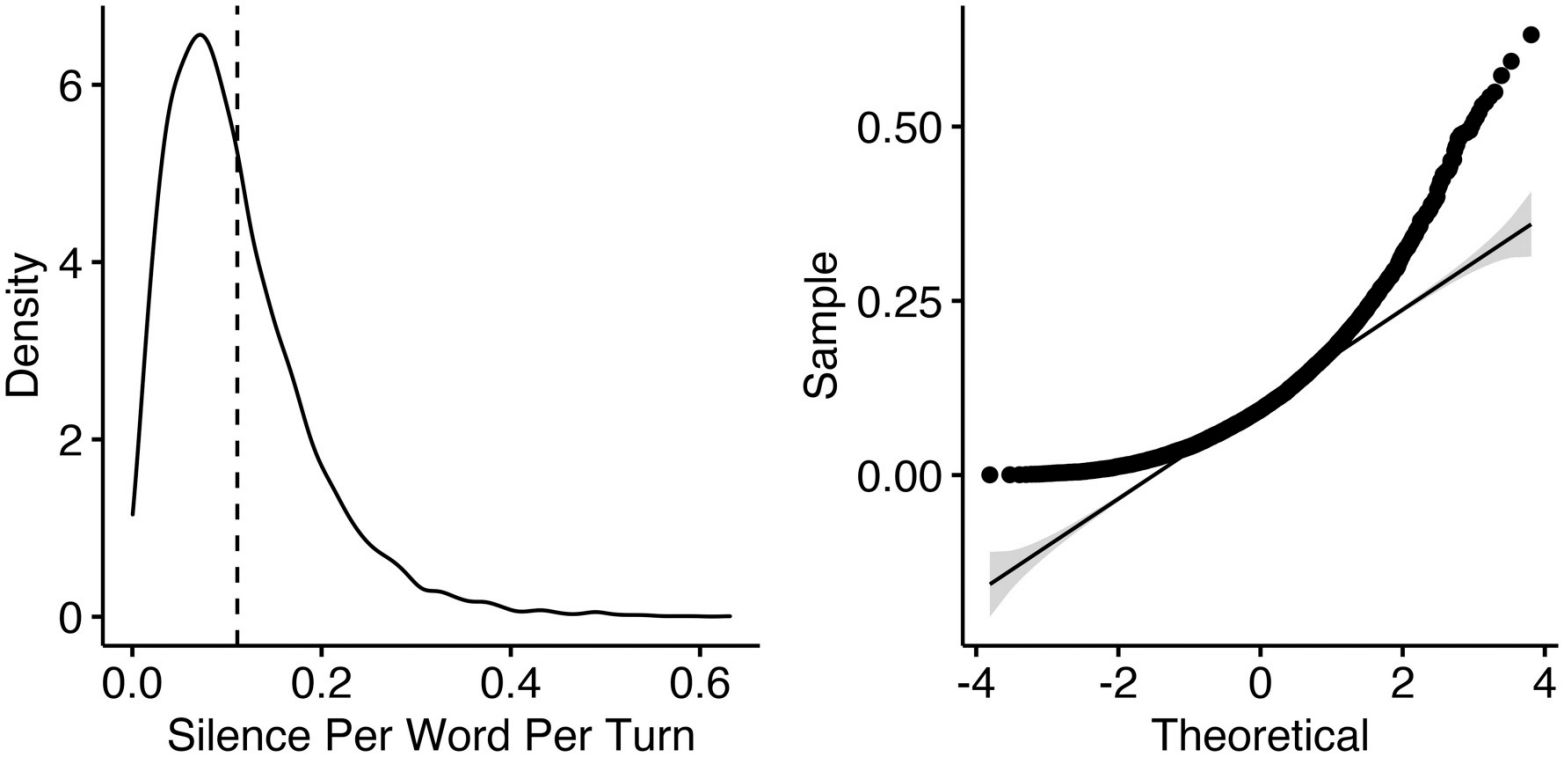
Density plot (left) and quartile-quartlile plot (right) of silence (s) per word per turn among young South Midland females in the Switchboard corpus.

Finally, for unfilled pauses, the mean amount of silence per word per turn is 0.11 ± 0.08 s, represented by the dashed line in Fig 3; however, as Fig 3 shows, the distribution of silence measurements is right-skewed. For this reason, in our linear regression below, we log-transform values to better meet the assumption of normality.

To determine effect directions we employ regression analysis. For the binary variable presence vs. absence of filled pauses we employ mixed-effects logistic regression using the *lme4* package in *R* [145, 146]. We also make use of the following additional packages: *car* [147]; *MuMin* [148]; *JGmermod* [149]. For the continuous variable of silence per word we employ mixed-effects linear regression, using the same package. In both regression analyses we include several additional control predictors known to influence disfluency (reported in Table 5), including overall turn length, speech rate (number of spoken words in a turn by the total duration of a turn), and content complexity, operationalized as mean orthographic character length of all words in a turn (under the assumption that the length of words reflects their conceptual complexity [150]). Each of these variables was centered and scaled by subtracting each value from the variable’s mean and dividing by the standard deviation.

**Table 5:** Mean observations of turn duration (s), mean word length (characters) by turn and speech rate (words/s) by turn among young South Midland women in the Switchboard Corpus.

Predictor	Mean	S.D.	Range
Turn duration	6.12 s	±3.31 s	1.16–14.00 s
Mean word length	3.64 characters	±0.56 characters	1.29–7.20 characters
Speech rate	3.22 words/s	±0.81 words/s	0.72–9.37 words/s

Before we present our regression analyses, it is important to note that we performed multiple parallel and often more complex analyses to ensure that our findings are robust in the face of various modeling decisions. These included regressions in which we treated both number of filled pauses and number of variable contexts as ordinal rather than binary. We also fitted models including the number of types of variable contexts per turn rather than overall number of variable contexts. We measured unfilled pauses in different ways, including testing total silence per turn, number of silences greater than a certain threshold per turn, and silence as a percentage of turn length. We tested random slopes in addition to random intercepts and included specific audio file as an additional random effect. These analyses required us to use a handful of alternative regression techniques (Poisson regression, negative-binomial regression, hurdle analyses). None of these parallel analyses revealed materially different patterns or statistically better fits to the data than those reported below. Following Occam’s razor we present these less complex regressions as, we contest, they are the most clearly illustrative of the phenomena under investigation.

Table 6 reports the mixed-effects logistic regression model testing the probability of at least one filled pause occurring in a turn. Fixed-effects include the presence of at least one variable context (reference level is *no variable contexts*), as well as the three predictors listed in Table 5, centered and scaled. Finally, we also include speaker as a random effect, as individual speakers contribute multiple observations to the data.

**Table 6:** Mixed-effects logistic regression testing the fixed effects of presence of variable contexts (reference level = *absent*), turn duration, mean word length and speech rate and the random intercept of speaker on the presence of filled pauses among young South Midland females in the Switchboard Corpus. Linear predictors centered and scaled. Treatment contrast coding. Model fit by maximum likelihood (Laplace Approximation). Model converges with BOBYQA optimizer with <20,000 iterations. Coefficients reported in log-odds. Variation inflation factors <1.34.

Observations: 7,161 (overall frequency of filled pauses 41%, *n*=2,934)				
Marginal *R*^2^ 0.22, Conditional *R*^2^ 0.31, Condition Number 2.75				
Fixed Effects	Coefficient	S.E.	*z*	Sig. Level
Intercept	-1.057	0.127	-8.413	***
Variable contexts	-0.096	0.067	-1.428	
Turn duration	0.979	0.356	27.511	***
Mean word length	-0.470	0.336	-13.992	***
Speech rate	-0.597	0.037	-16.098	***
Random Effects	Variance		*N*	
Speaker (Intercept)	0.447		34	

*p<0.05;

**p<0.01;

***p<0.001

Table 6 shows that when length of turn, mean word length, and speech rate are controlled, the effect of overall presence of variable contexts is non-significant. Unsurprisingly, given the results reported by Shriberg [26, 22], the effect of turn duration is significant and positive. Longer turns are more likely to have filled pauses. Mean word length and speech rate, on the other hand, have negative effects. *Um’s* and *uh’s* are more likely when speakers are speaking slowly, or when they are using (on average) shorter words. Conversely, overt disfluencies are less likely when speakers are speaking quickly and using, on average, longer words. Both findings are somewhat counter-intuitive. But note that causality is unclear: rather than slow speech and usage of shorter words triggering disfluencies, perhaps disfluencies coincide with slow speech and usage of shorter words (cf. [151]).

Though non-significant, the effect of variable context is also negative, suggesting that there are fewer filled pauses in turns in which variable contexts occur. But what about individual linguistic variables? We hypothesized that highly salient linguistic variables or alternations with overt style prescriptions would coincide with higher rates of disfluency. In our list of 20 variables these were quotatives and coordinated pronouns. As Table 7 shows, neither of these variables are significant predictors of the presence of filled pauses. When all variables were included as fixed effects, three individual variables were identified as significant: expressions of future temporal reference, of expressions of deontic modality, and *not* vs. *no* negation. The effect for these too was negative, indicating the presence of these variable contexts decreases the likelihood of a filled pause.

**Table 7:** Mixed-effects logistic regression testing the fixed effects of the binary presence of 20 linguistic variables (reference level = *absent*), turn duration, mean word length and speech rate and the random intercept of speaker on the presence of filled pauses among young South Midland females in the Switchboard Corpus. Linear predictors centered and scaled. Treatment contrast coding. Model fit by maximum likelihood (Laplace Approximation). Model converges with BOBYQA optimizer with <20,000 iterations. Coefficients reported in log-odds. Variation inflation factors <1.43.

Observations: 7,161 (overall frequency of filled pauses 41%, *n*=2,934)						
Marginal *R*^2^ 0.23, Conditional *R*^2^ 0.32, Condition Number 2.78						
Fixed Effects	Coefficient	S.E.	*z*	Sig. Level
Intercept	-1.002	0.124	-8.165	***
Indefinite pronouns	-0.315	0.176	-1.792	
Coordinated pronouns	-0.013	0.372	-0.036	
*That* vs. zero complementizers	-0.164	0.093	-1.757	
Infinitival vs. gerundial complementation	0.092	0.369	0.250	
*Remember*, *regret*, *deny* complementation	0.385	0.530	0.726	
*Try to* vs. *try and* vs. *try -ing*	-0.064	0.353	-0.181	
*Tried to* vs. *try -ing*	0.429	0.657	0.652	
Particle placement	-0.328	0.173	-1.900	
Dative alternation	-0.156	0.420	-0.370	
Genitive alternation	0.0005	0.079	0.007	
Restrictive relativizers	-0.054	0.164	-0.330	
Nonrestrictive relativizers	-0.008	0.182	-0.044	
Analytic vs. synthetic comparatives	0.033	0.451	0.074	
*There is*/*was* with plural subjects	-0.111	0.182	-0.044	
Expressions of future temporal reference	-0.418	0.124	-3.358	***
Expressions of deontic modality	-0.352	0.156	-2.261	*
Expressions of stative possession	0.085	0.117	0.730	
Quotatives	-0.496	0.269	-1.848	
*Not* vs. *no* negation	-0.410	0.187	-2.190	*
*Not* vs. auxiliary contraction	-0.038	0.120	-0.314	
Turn duration	1.018	0.037	27.563	***
Mean word length	-0.474	0.034	-13.937	***
Speech rate	-0.568	0.037	-15.237	***
Random Effects	Variance		*N*	
Speaker (Intercept)	0.441		34	

*p<0.05;

**p<0.01;

***p<0.001

**Fig 4:**
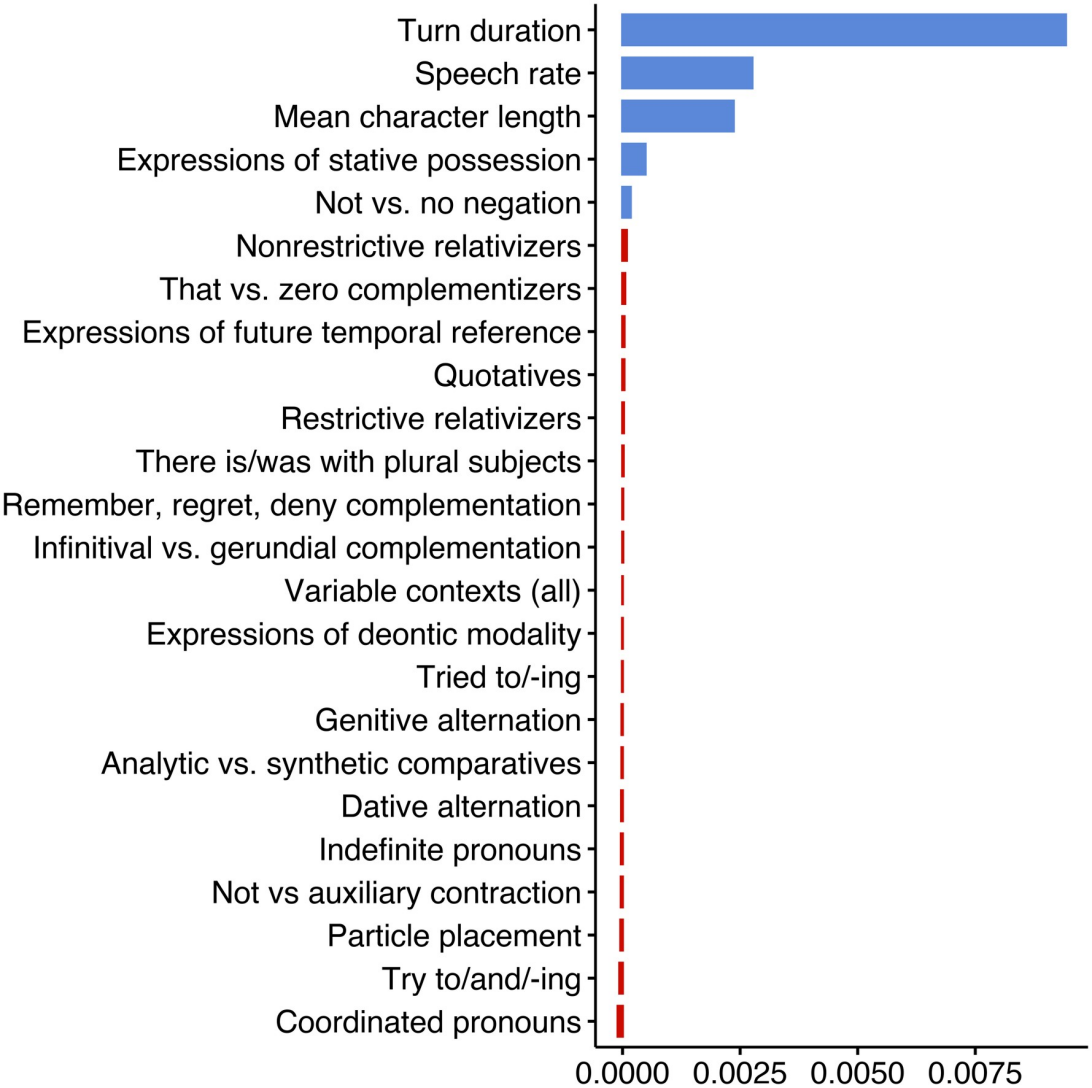
Conditional permutation variable importance of a random forest of the binary presence of a filled pause in a turn among young South Midland females in the Switchboard Corpus. Model parameters: 500 trees; 4 randomly-preselected variables for each split. Seed displayed: 2,000. Model accuracy: 82%: kappa: 0.48; *C*-index/AUROC: 0.72.

To determine the relative importance of predictors we subject the data to random forest analysis as implemented by the *randomForest* package in *R* [152]. This algorithm evaluates the success of (here 500) randomly-built classification trees at predicting the variation in the data. Random forests are especially useful because they can simultaneously test predictors that are non-orthogonal, which, by definition, individual linguistic predictors and the overall number of linguistic predictors must be [153, 154]. We build our random forest using the same fixed effect predictors presented in Tables 6 and 7 (in other words, the random forest formula includes the three control predictors, all linguistics variables, plus the binary presence of any linguistic variable). The number of randomly pre-selected variables to start each tree in the forest, 4, was selected based on minimum out-of-bag error calculated using the *tuneRF()* function in the *randomForest* package. The relative importance (i.e. success), of each predictor was determined using the *permimp* [155] package. Fig 4 presents these values. Parameters with variable importance greater than the absolute value of the maximum negative variable importance (indicated by blue bars) are considered successful predictors. Three performance metrics: accuracy, Kappa and *C*-index/AUROC, were calculated using the *caret* [156] and *ROCR* [157] packages. The accuracy metric, based on the confusion matrix, indicates that the random forest correctly predicts 82% of the variation. The kappa statistic, which evaluates agreement between observed and expected accuracy, is 0.48, or moderate [158, 165]. A *C*-index/AUROC (area under the receiver-operator curve) of 0.72 is considered acceptable discrimination [159, 162] [160, 259].

Fig 4 shows that turn duration has the highest variable importance, followed by speech rate and mean character length. These control variables were the three significant predictors identified in Table 6. To a much lesser degree, the presence of expressions of stative possession and *not* vs. *no* negation are successful predictors. The former was not identified in Table 7. The other significant predictors in Table 7, expression of future temporal reference and expressions of deontic modality, were not determined to be successful predictors in the random forest. The mismatch between the regression and random forest analysis, plus the relatively low importance of stative possession and *not* vs. *no* negation in the random forest, suggests that while these predictors may be correlated to the presence of filled pauses, they do not explain much of the variation. Of note, when additional forests were constructed using different sets of randomly generated trees (i.e., by manipulating R’s random number generating function *set.seed()*), the three control variables were consistently top ranked and in the same order, while anywhere from one to nine different linguistic variables showed some small, randomly ordered importance. In no tree was the overall number of variable contexts a successful predictor. This is far from the significant positive correlation we would expect to find if the presence of linguistic variation truly increased cognitive load.

There is a *prima facie* intuition that the longer a turn, the more potential there is for a variable context to occur—in other words, that these two predictors are collinear. But this is not necessarily the case. The variable inflation factors for Table 6 are all less than <1.34, indicating very little multicollinearity among input parameters to the regression model. Further the condition number is low (2.75), also indicating little multicollinearity. Finally, the *find.interaction()* function in the *randomForestSRC* package [161] (see also [154, 24]), which finds pairwise interactions between predictors in a random forest did not identify any substantial interaction between turn duration and presence of a variable context (or any other two predictors). With this proviso, it is still informative to explore the relationship between these two predictors on the realization of filled pauses.

**Fig 5:**
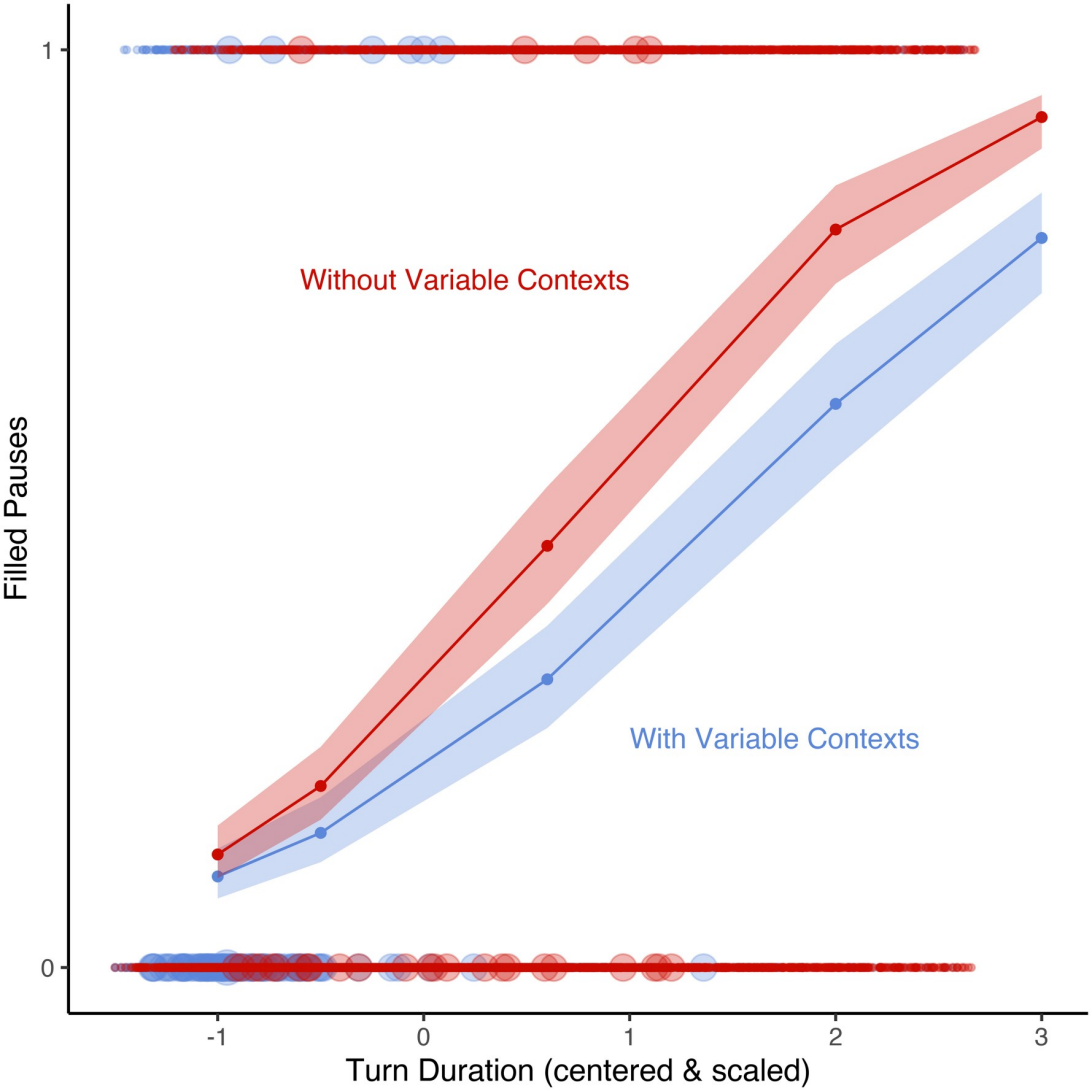
Effects plot of estimate of presence of a filled pause by turn duration for two regression models fitted with turn duration, mean word length, and speech rate as fixed predictors, and speaker as a random effect, for all turns containing a variable context (blue line) and not containing a variable context (red line) among young South Midland speakers in the Switchboard Corpus. Points at 1 and 0 on the y-axis represent the actual observed values of filled pauses (1 = present), (0 = absent). Curves represent fitted probabilities.

Fig 5 plots the estimates of turn duration for regression models (with turn duration, mean word length, and speech rate as fixed predictors, and speaker as a random effect) containing only data in which at least one variable context is present (blue) and only data in which there are no variable contexts (red). It shows that the effect of turn duration, whereby a filled pause is increasingly more likely the longer the turn, is attenuated by the presence of a variable context. Not only are variable contexts not positively correlated with filled pauses, but in fact they act to reduce the effect that longer turn duration has on the realization of a filled pause. This is absolutely not consistent with the view that variables contexts cause increased disfluency.

Tables 8 and 9 present the mixed-effects linear regression model testing the effect of the same predictors discussed above on the length of silence per word in each turn. As with filled pauses, mean word length and speech rate are inversely related to length of silence per word. With overall longer words in a turn and with faster speaking the length of silence per word decreases. Again, these effect directions are surprising, but causality remains unclear—though Engelhardt *et al.* [151] did find that children and adults who scored high on the vocabulary portion of the Weschler Intelligence Scale tests produced fewer unfilled pauses in a laboratory experiment.

**Table 8:** Mixed-effects linear regression testing the fixed effects of presence of variable contexts (reference level = *absent*), turn duration, mean word length and speech rate and the random intercept of speaker on length of speech planning time per turn among young South Midland females in the Switchboard Corpus. Speech planning time log transformed. Linear predictors centered and scaled. Treatment contrast coding. Model fit by maximum likelihood. Probability calculated using Satterthwaite approximation for degrees of freedom. Coefficients reported in log-odds. Variation inflation factors <1.23.

Observations: 7,161 (silence per turn Mean = 111 ms, SD = ±78 ms)					
Marginal *R*^2^ 0.23, Conditional *R*^2^ 0.41, Condition Number 2.75					
Fixed Effects	Coefficient	S.E.	df	*t*	Sig. Level
Intercept	-2.431	0.060	34.98	-40.401	***
Variable Contexts	-0.011	0.016	7,135	-0.673	
Duration	-0.034	0.008	7,143	-4.142	***
Mean Word Length	-0.066	0.007	7,133	-8.599	***
Speech Rate	-0.382	0.008	7,149	-45.825	***
Random Effects	Variance			*N*	
Speaker(Intercept)	0.1179			34	

**Table 9:** Mixed-effects linear regression testing the fixed effects of presence of variable contexts (reference level = *absent*), turn duration, mean word length and speech rate and the random intercept of speaker on length of speech planning time per turn among young South Midland females in the Switchboard Corpus. Speech planning time log transformed. Linear predictors centered and scaled. Treatment contrast coding. Model fit by maximum likelihood. t-tests use Satterthwaite’s method. Coefficients reported in log-odds. Variation inflation factors <1.29.

Observations: 7,161 (silence per turn M = 111 ms, SD = ±78 ms)					
Marginal *R*^2^ 0.23, Conditional *R*^2^ 0.42, Condition Number 2.78					
Fixed Effects	Coefficient	S.E.	df	*t*	Sig. Level
Intercept	-2.434	0.060	34.66	-40.718	***
Indefinite pronouns	-0.092	0.042	7,130	-2.166	*
Coordinated pronouns	-0.199	0.098	7,130	-2.031	*
*That* vs. zero complementizers	0.025	0.028	7,132	1.078	
Infinitival vs. gerundial complementation	-0.065	0.091	7,130	-0.717	
*Remember*, *regret*, *deny* complementation	-0.476	0.141	7,128	-3.373	***
*Try to* vs. *try and* vs. *try -ing*	-0.016	0.093	7,129	-0.170	
*Tried to* vs. *try -ing*	0.005	0.164	7,128	0.036	
Particle placement	-0.029	0.043	7,129	-0.671	
Dative alternation	0.0695	0.106	7,129	0.658	
Genitive alternation	-0.018	0.020	7,131	-0.910	
Restrictive relativizers	0.066	0.043	7,133	1.551	
Nonrestrictive relativizers	0.128	0.047	7,133	2.730	**
Analytic vs. synthetic comparatives	-0.169	0.161	7,130	-1.455	
*There is*/*was* with plural subjects	0.120	0.088	7,130	1.359	
Expressions of future temporal reference	0.056	0.030	7,130	1.892	
Expressions of deontic modality	0.015	0.037	7,130	0.421	
Expressions of stative possession	-0.002	0.029	7,133	-0.077	
Quotatives	-0.227	0.061	7,131	-3.735	***
*Not* vs. *no* negation	-0.110	0.046	7,129	-2.386	*
*Not* vs. auxiliary contraction	-0.006	0.030	7,129	-0.198	
Turn duration	-0.034	0.009	7,144	-4.044	***
Mean word length	-0.065	0.008	7,133	-8.466	***
Speech rate	-0.382	0.008	7,148	-45.677	***
Random Effects	Variance			*N*	
Speaker(Intercept)	0.1168			34	

**Fig 6:**
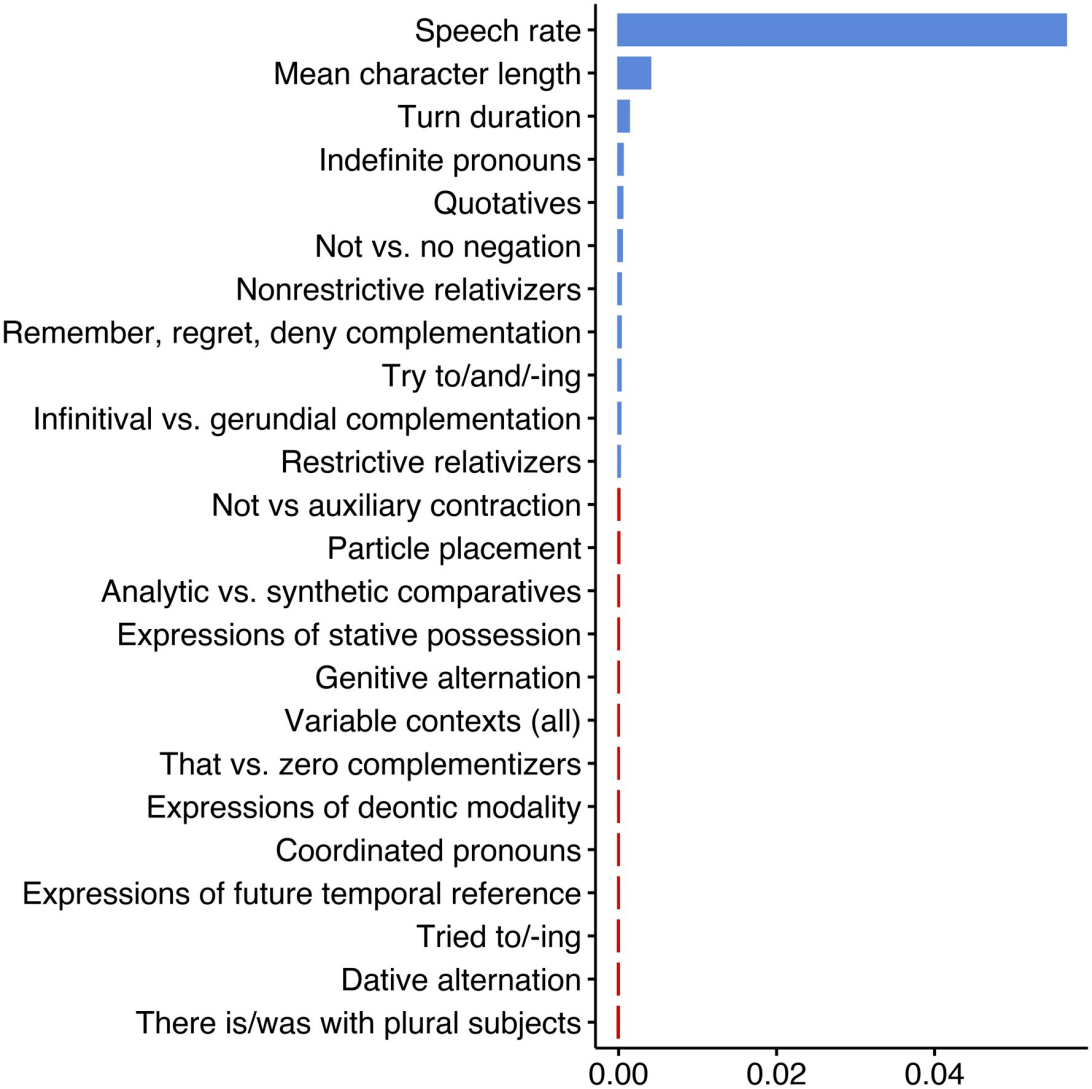
Conditional permutation variable importance of a random forest of the amount of silence per word in a turn among young South Midland females in the Switchboard Corpus. Model parameters: 500 trees; 6 randomly-preselected variables for each split. Seed displayed: 2,000. Model Root Mean Square Error (RMSE):0.71.

Unlike filled pauses, duration has a negative effect for silence. In longer turns, silence per word is shorter, while in shorter turns, silence per word is longer. The *lme4* package in *R* no longer generates *p*-values to assess the significance of predictors in linear regression (see [162, 4, 163, 247–248, 164]). To attain *p*-values we follow the package authors’ advice and use the *lmerTest* package [165] to compute *t*-tests using the Satterthwaite approximation for degrees of freedom. Finally, in Table 8 the binary presence of a variable context has a negative effect, though it is not significant. In Table 9 there are several significant linguistic predictors and all but one (nonrestrictive relativizers) have a negative effect. The negative effect predictors are indefinite and coordinated pronouns, *remember, regret,* and *deny* complementation, quotatives, and *not* vs. *no* negation. When we look at the random forest testing all predictors (Fig 6), however, we see that, relative to speech rate, and, to a lesser extent, mean character length, these linguistic predictors, though significant, offer minor explanatory power. Again, as with filled pauses, we do not see a significant positive effect of the presence of variable contexts on the realization of silence in a turn. How much pausing occurs between words appears to be largely governed by how quickly someone is speaking, to a small extent by how long the words in the turn are, and meaningfully by very little else. Where variation occurs it also almost always coincides with less silence, not more.

It is notable that the two variables we hypothesized above to require additional extra-grammatical consideration (i.e., more processing triggered by the self-monitoring mechanism), the rapidly innovating (in the 1990s) variable (quotatives) and the variable with perhaps the most salient, overt prescription (coordinated pronouns)—entailing both morphological and linear order prescriptions—showed either no significant relationship or a significant negative relationship to speech production difficulties. In fact, both correspond to shorter unfilled pauses and to the absence of filled pauses, defying expectations. That being said, the one linguistic feature with a positive effect, nonrestrictive relativizers, is the variable for which some linguists do not believe that variation exists (e.g., [4], though see 14).

## Discussion

According to the literature, there is a clear link between increased disfluency/speech planning time and increased cognitive load, caused by complex grammatical structures, utterance length, discursive task, exerting executive control over speech planning, etc. (discussed above). If the processing of variation adds to this cognitive load it is expected that there would be a positive correlation between disfluency/speech planning time and variable contexts. In fact, this correlation should be significant and strong. Speakers must check the linguistic context for conditioning factors and make complex probabilistic computations to select a grammatical variant—and in contexts of style-shifting or audience design this process additionally exploits an executively-controlled self-monitoring process. Prior to the current investigation the prevailing thought was that this surely must add to cognitive load. Instead, what our investigation has shown is that the processing of the complex probabilistic constraints needed to select a specific grammatical variant does not result in significant measurable production difficulty using two separate metrics: filled and unfilled pauses. Our results directly challenge the intuition that variation must be difficult and complex from a production standpoint. We find that overall the presence of variation poses no measurable effect. With the exception of nonrestrictive relative clauses and unfilled pauses, for the 20 variables under scrutiny, the relationship between the presence of variation and filled pauses and/or length of unfilled pauses is either non-significant, or significant and negative.

As Pouget *et al.* [166] point out, probabilistic reasoning is not atypical for the brain. Experimental work over the last 30 years has shown that human behavior is highly consistent with probabilistic reasoning (or sampling [167]) not only in the sensory domain [168–172], but also in the motor [173–175] and cognitive [176–181] domains. It would be atypical if the brain could not plan and self-monitor easily based on probabilistic constraints. The presence of grammatical variability, from this point of view, should not lead to greater processing difficulty.

While we remain agnostic to the precise nature of the mechanism that makes probabilistic constraint-based variant selections in the grammatical encoding process, we propose that this probabilistic computation of variant alternatives is largely automatic. Further, this automatic computation process does not seek out loci of variation but instead evaluates all grammatical structures. This computation must occur for all units within an utterance. That is, some form of computation must occur when planning every aspect of an utterance at all levels of grammar (lexical retrieval, phonetics, phonology, prosody, etc.). Likewise, the self-monitoring process must monitor (and potentially substitute/repair) units at every level of grammar (following [47], though see [49, 50, 52]). This perhaps aligns better with a cognitive network model of language production as described by Thomas [182] and others in which sociolinguistic knowledge is integrated with every structural element of the grammar. In this way, the potential for variation is ubiquitous—and if any production difficulty is caused by selecting variants, that difficulty has the potential to arise both where grammatical variation exists and where it does not.

It may further be the case that, in certain contexts, the availability of multiple variants actually facilitates production, offsetting any potential planning difficulty caused by ‘choice’. The analogy is that hitting any target increases when the number of targets on the firing range is increased. This extends Christenfeld’s [45] notion of lexical suppression to the full utterance; fewer potential well-formed versions of an utterance increases the difficulty in effectively planning and implementing a well-formed utterance. Similarly, within an exemplar model of grammar (e.g., [183]), the presence of multiple possible exemplars for an intended meaning means the speaker has greater flexibility in selecting a well-formed utterance. Our findings are a direct challenge to Myachykov *et al.* [46] who conclude after their cross-linguistic study of Russian and English that syntactic flexibility (i.e., grammatical variation) is cognitively costly.

Our findings, however, do align exactly with the conclusions of Engelhardt *et al.* [184]. In their laboratory experiment the authors required participants to generate sentences based on either unambiguous participles (like *ridden*) or ambiguous past tense/past participle forms (e.g., *-ed* forms of regular weak verbs like *dropped*) and two pictured objects (one animate, the other inanimate). Participants were more likely to make mistakes (i.e., produce ungrammatical sentences) with unambiguous participles, which the authors conclude was due to participle verbs being licit in fewer syntactic structures—unlike the ambiguous forms, unambiguous participles cannot be used for active simple past constructions. Flexibility in the production process facilitated achieving well-formedness.

Similarly, Ferreira [185] found experimentally that sentences headed by verbs that permit the dative alternation (like *give*) were produced quicker and more fluently than sentences with verbs that only allow the prepositional dative (like *donate*). He argues that speakers produce sentences more easily under conditions of syntactic flexibility because it allows the system to accommodate potential differences in activation states for the words involved over time (e.g., if the indirect object is activated before the direct object the double object dative construction can be enacted without production breaking down. As Ferreira & Engelhardt [186, 79] state, “one benefit of syntactic freedom of choice is that it enhances the efficiency of language production.”

Moreover, the putative difficulties introduced by optionality in syntactic structure or morphological realization are likely offset by additional phenomena. Below we list five.

1. Rohdenburg [187, 149] notes that “more explicit grammatical alternatives tend to be preferred in cognitively more complex environments”. Building on this idea, we suggest that having the option to use an overt complementizer (e.g., *that*) can facilitate the planning of otherwise complex (and thus difficult to plan) complements (see [186] for a review of supporting studies). Alternatively, having the option of a zero complementizer in simple utterances, where the existence of a complementizer is predictable, facilitates communicative efficiency, another desideratum.
2. The Uniform Information Density hypothesis [181, 188, 189] predicts that within the bounds defined by grammar, speakers prefer utterances that distribute information uniformly across the signal (information density). Where speakers have a choice between several variants to encode their message, they prefer the variant which accomplishes more uniform information density. Variation between zero and realized variants (like complementizer *that* or quotative *say*) or between shorter/ambiguous variants (*’s*,*’ll*) and longer/overt variants (*’s got*, *’s going to*) provides flexibility to the speaker in spreading out information density (i.e., to make it more uniform) when following material (or even preceding material) is otherwise too information dense.
3. Gries [91] reports that the preferred ordering for particle verbs with simple direct objects is the split pattern; however, as the direct object becomes more complex (expressed with more words), the preference changes to the joined pattern. This tendency is reinforced by the fact that new information (itself requiring more processing effort) is usually encoded using more material [91, 103], so that longer direct objects are doubly difficult to process. Lohse *et al.* [93] argue that Gries’ [91] observations can be explained by domain minimization, whereby the human processor prefers to minimize the connected sequences of linguistic forms and their conventionally associated syntactic and semantic properties in which relations of combination and/or dependencies are processed. In other words, the longer the direct object, the more likely the joined pattern will be, as the split particle would otherwise be increasingly further away from the verb. The joined pattern is also preferred for idiomatic particle verbs because there is a strong semantic dependency between the verb and particle (see also [190]). Regardless, under both Gries’ and Lohse *et al.*’s explanation, having the option to express the particle in different locations allows the speaker to reduce production difficulty as needed.
4. Arnold *et al.* [191] report for the Switchboard corpus that already known or ‘given’ material and less complex or ‘light’ material, which are easier to process, are produced earlier in utterances. There is a universal tendency across languages for both ‘heavy’ and ‘new’ material to occur later in an utterance (see review in [191]). Just like given and light constituents, animate constituents also tend to occur before inanimate constituents in an utterance (see [192] and the review therein). The option to adhere to these these tendencies likely makes planning utterances containing genitives or ditransitive datives, in which these tendencies can be met, easier than utterances where violating these tendencies is unavoidable for well-formedness.
5. Finally, syntactic or morphological optionality may facilitate planning as it can provide the needed phonological material for a desired prosodic pattern of the utterance (e.g., the rhythmic alternation between stressed and unstressed syllables). For example, Shih *et al.* [102] find eurythmicity to be a significant predictor of different genitive variants. Anttila *et al.* [193] find the same for the dative alternation using data from the Switchboard Corpus (see, again, [186] for a survey of supporting research).

A limitation of our analysis is that our metrics of processing difficulty are perhaps unreliable, as disfluent phenomena can serve both discourse functions as well as indicate processing difficulties on behalf of the speaker. The former is called the signal hypothesis, the latter the symptom hypothesis (the starting point for the present study). The signal hypothesis interprets disfluencies like *um* and *uh* as tools speakers use to signal something (e.g., delay or new/complex upcoming information, metacognitive status, structural boundaries) to the listener [61, 194–196]. Likewise, silence can signal syntactic structure, utterance boundaries, rhetorical and expressive emphasis, or stylistic/identity peculiarities [68, 197]. Of course, neither hypothesis need exclude the other. Further, it is reasonable to assume that non-disfluent “disfluencies” and structural/stylistic silence would be evenly distributed across multiple turns so that in the aggregate their use is neutralized. We are therefore confident that our findings do support the conclusion that variation is not measurably more difficult to produce.

Perhaps our most surprising finding is that presence of a quotative verb does not correspond to the presence of filled pauses or longer speech planning time. This challenges the hypotheses that socially-salient variables—like quotatives in the 1990s (e.g., [198]) given the rise of innovative *be like* among young speakers—would be most likely to coincide with production difficulties because the self-monitoring process must evaluate them in light of both the linguistic and social context. The nature of how quotatives are used, however, may explain this finding. Generally direct quotation is associated with narrative [199], a type of speech that is particularly vernacular—and thus less monitored. For this reason turns with quotative verbs may be, more often than not, narrative-style speech, which is less self-monitored, and therefore less likely to be disfluent.

## Conclusion

Our point of departure in this paper was the suspicion among both language mavens and (some) professional linguists that variation is unexpected, suboptimal, (needlessly) complex, and difficult for language users. But our analysis shows that among young South Midland females, the thusly expected significant positive correlation between the presence of grammatical variable contexts and two metrics of processing difficulty, filled and unfilled pauses, simply cannot be established statistically. We have suggested that the general mechanism for probabilistic variable rule implementation must monitor all components of an utterance, and thus morphosyntactic loci of variation do not uniquely result in any additional burden. Further we have offered several suggestions for why the presence of optionality may actually offset putative production difficulties, mainly through providing flexibility to the speaker in signalling syntactic structure, maximizing efficiency, spreading out information density, minimizing distance between dependent elements, adhering to universal tendencies, and/or aligning syntax to prosody.

Given these results, we aim to discover in future enquiries if this pattern persists among men or across different age groups, topics, or American dialect regions in the full Switchboard Corpus. Moving forward we will need to employ additional statistical tests to asses the effect of age, sex, region, education, topic, etc. on this relationship. As to grammatical variation in particular, future work should also investigate if grammatical variables differ in the extent to which they attract or repel disfluencies as a function of the number of probabilistic constraints by which they are conditioned, or as a function of the number of variants that they include. It would also be desirable to include surprisal/entropy and lexical access measures as controls in models predicting disfluencies, and to further consider the exact placement of disfluencies (i.e. before or after grammatical variation contexts). Finally, our analysis should—needless to say—also be extended to lexical and phonological variables (given [47]).

## Supporting information

S1 Data (TXT)

S2 Data (HTML)
