# Supplementary material for: Variation isn’t that hard: Morphosyntactic choice does not predict production difficulty
Source: PLoS One. 2021 Jun 21;16(6):e0252602. doi: 10.1371/journal.pone.0252602 (PMC8216537; doi:10.1371/journal.pone.0252602)
Supplement: S2 Data — (HTML) [file pone.0252602.s002.html]

Variation isn’t that hard: Morphosyntactic choice does not predit production difficulty


# Variation isn’t that hard: Morphosyntactic choice does not predit production difficulty

#### Gardner, Uffing, Van Vaek, & Szmrecsanyi

#### 06/05/2021

# What is Reported in the Manuscript

The following code reproduces the figures and simplified models presented in our manuscript.

```
library(tidyr)
library(dplyr)
library(lme4)
library(car)
library(MuMIn)
library(JGmermod)
library(randomForest)
library(permimp)
library(ggpubr)
library(caret)
library(ROCR)
library(randomForestSRC)
library(lmerTest)
library(effects)
```

```
df<-read.delim("MHGetal-Datafile.txt")
```

```
df<-df%>%
  filter(Audiofile != "3248" & #1 turn
         Audiofile != "4157" & #1 turn
         Audiofile != "2597" & #2 turn
         Audiofile != "4149" & #2 turn
        WordPerSec < 7.6 & # 3 turns
          Turn.Duration < 17) # 1 turn
```

```
df<-df%>%
  mutate(NVarbs.bi = factor(ifelse(NVarbs > 0, "1", "0")),
         NFP.bi = factor(ifelse(NFP > 0, "1", "0")),
         V01.bi = factor(ifelse(V01 > 0, "1", "0")),
         V02.bi = factor(ifelse(V02 > 0, "1", "0")),
         V03.bi = factor(ifelse(V03 > 0, "1", "0")),
         V04.bi = factor(ifelse(V04 > 0, "1", "0")),
         V05.bi = factor(ifelse(V05 > 0, "1", "0")),
         V06.bi = factor(ifelse(V06 > 0, "1", "0")),
         V07.bi = factor(ifelse(V07 > 0, "1", "0")),
         V08.bi = factor(ifelse(V08 > 0, "1", "0")),
         V09.bi = factor(ifelse(V09 > 0, "1", "0")),
         V10.bi = factor(ifelse(V10 > 0, "1", "0")),
         V11.bi = factor(ifelse(V11 > 0, "1", "0")),
         V12.bi = factor(ifelse(V12 > 0, "1", "0")),
         V13.bi = factor(ifelse(V13 > 0, "1", "0")),
         V14.bi = factor(ifelse(V14 > 0, "1", "0")),
         V15.bi = factor(ifelse(V15 > 0, "1", "0")),
         V16.bi = factor(ifelse(V16 > 0, "1", "0")),
         V17.bi = factor(ifelse(V17 > 0, "1", "0")),
         V18.bi = factor(ifelse(V18 > 0, "1", "0")),
         V19.bi = factor(ifelse(V19 > 0, "1", "0")),
         V20.bi = factor(ifelse(V20 > 0, "1", "0")),
         V01.C = as.numeric(scale(V01, scale=TRUE)),
         V02.C = as.numeric(scale(V02, scale=TRUE)),
         V03.C = as.numeric(scale(V03, scale=TRUE)),
         V04.C = as.numeric(scale(V04, scale=TRUE)),
         V05.C = as.numeric(scale(V05, scale=TRUE)),
         V06.C = as.numeric(scale(V06, scale=TRUE)),
         V07.C = as.numeric(scale(V07, scale=TRUE)),
         V08.C = as.numeric(scale(V08, scale=TRUE)),
         V09.C = as.numeric(scale(V09, scale=TRUE)),
         V10.C = as.numeric(scale(V10, scale=TRUE)),
         V11.C = as.numeric(scale(V11, scale=TRUE)),
         V12.C = as.numeric(scale(V12, scale=TRUE)),
         V13.C = as.numeric(scale(V13, scale=TRUE)),
         V14.C = as.numeric(scale(V14, scale=TRUE)),
         V15.C = as.numeric(scale(V15, scale=TRUE)),
         V16.C = as.numeric(scale(V16, scale=TRUE)),
         V17.C = as.numeric(scale(V17, scale=TRUE)),
         V18.C = as.numeric(scale(V18, scale=TRUE)),
         V19.C = as.numeric(scale(V19, scale=TRUE)),
         V20.C = as.numeric(scale(V20, scale=TRUE)),
         Turn.Duration.C=as.numeric(scale(Turn.Duration, scale=TRUE)),
         CharPerWord.C=as.numeric(scale(CharPerWord, scale=TRUE)),
         WordPerSec.C=as.numeric(scale(WordPerSec, scale=TRUE)),
         SilPerWord.log = log(SilPerWord))
```

```
df%>%
  summarise(Total.Tokens =n(), Audiofile=n_distinct(Audiofile),Speaker_Number=n_distinct(Speaker_Number), Variable_Contexts=sum(NVarbs), Filled_Pauses=sum(NFP), Silence.Mean = mean(SilPerWord, na.rm=TRUE), Silence.SD = sd(SilPerWord))
```

```
##   Total.Tokens Audiofile Speaker_Number Variable_Contexts Filled_Pauses
## 1         7161       285             34              6268          2934
##   Silence.Mean Silence.SD
## 1     0.110922 0.07754336
```

```
df%>%
  count(NVarbs)%>%
  mutate(prop=prop.table(n))
```

```
##    NVarbs    n         prop
## 1       0 3693 0.5157100964
## 2       1 1879 0.2623935205
## 3       2  897 0.1252618349
## 4       3  388 0.0541823768
## 5       4  181 0.0252757995
## 6       5   67 0.0093562352
## 7       6   39 0.0054461667
## 8       7    8 0.0011171624
## 9       8    3 0.0004189359
## 10      9    3 0.0004189359
## 11     10    2 0.0002792906
## 12     11    1 0.0001396453
```

```
df%>%
  count(NFP)%>%
  mutate(prop=prop.table(n))
```

```
##   NFP    n         prop
## 1   0 5000 0.6982265047
## 2   1 1559 0.2177070242
## 3   2  461 0.0643764837
## 4   3  115 0.0160592096
## 5   4   22 0.0030721966
## 6   5    4 0.0005585812
```

```
options(contrasts=c("contr.treatment","contr.poly"))
```

```
dens<-ggdensity(df$SilPerWord,
  xlab = "Silence Per Word Per Turn",
  add = "mean",
  ylab="Density"
)
qq<-ggqqplot(df$SilPerWord)

figure<-ggarrange(dens, qq,
                ncol=2, 
                nrow=1)
figure
```

```
glmer.FP <-glmer(NFP.bi ~ NVarbs.bi + Turn.Duration.C + CharPerWord.C + WordPerSec.C + (1 |Speaker_Number), data=df, family="binomial", control = glmerControl(optCtrl =list(maxfun = 2e4), optimizer = "bobyqa"))
summary(glmer.FP)
r.squaredGLMM(glmer.FP)
collin.fnc.mer(glmer.FP)$cnumber
vif(glmer.FP)


glmer.FP.all<-glmer(NFP.bi~V01.bi+V02.bi+V03.bi+V04.bi+V05.bi+V06.bi+V07.bi+V08.bi+V09.bi+V10.bi+V11.bi+V12.bi+V13.bi+V14.bi+V15.bi+V16.bi+V17.bi+V18.bi+V19.bi+V20.bi+Turn.Duration.C+ CharPerWord.C+ WordPerSec.C+(1|Speaker_Number), data=df, family="binomial", control = glmerControl(optCtrl =list(maxfun = 2e4), optimizer = "bobyqa"))
summary(glmer.FP.all)
r.squaredGLMM(glmer.FP.all)
collin.fnc.mer(glmer.FP.all)$cnumber
vif(glmer.FP.all)
```

```
df2<-df%>%
dplyr:: select(NFP.bi,V01.bi,V02.bi,V03.bi,V04.bi,V05.bi,V06.bi,V07.bi,V08.bi,V09.bi,V10.bi,V11.bi,V12.bi,V13.bi,V14.bi,V15.bi,V16.bi,V17.bi,V18.bi,V19.bi,V20.bi,NVarbs.bi,Turn.Duration.C,CharPerWord.C,WordPerSec.C)

set.seed(2000)
mtry<-tuneRF(df2[-1], df2$NFP.bi, ntreeTry=128, stepFactor=1.35, improve=0.01, trace=FALSE, plot=FALSE)

best.m<-min(mtry[mtry[,2] == min(mtry[,2]),1])

df.rf<-randomForest(NFP.bi~.,data=df2, ntree=500, mtry=best.m, replace=FALSE, keep.forest=TRUE, keep.inbag=TRUE, importance=TRUE, proximity=TRUE)

df.res<-permimp(df.rf, conditional=TRUE, progressBar=FALSE, do_check=FALSE)

df.res2<-gather(data.frame(as.list(df.res$values)), variable, value)

fullname<-c("Particle placement", "Dative alternation", "Genitive alternation", "Analytic vs. synthetic comparatives", "That vs. zero complementizers", "Infinitival vs. gerundial complementation", "Remember, regret, deny complementation","Expressions of future temporal reference", "Expressions of deontic modality", "Expressions of stative possession", "Restrictive relativizers", "Not vs. no negation", "Not vs auxiliary contraction", "Indefinite pronouns", "Coordinated pronouns", "Quotatives", "Try to/and/-ing", "Tried to/-ing", "There is/was with plural subjects", "Nonrestrictive relativizers", "Variable contexts (all)", "Turn duration", "Mean character length", "Speech rate")

variable<-c("V01.bi","V02.bi","V03.bi","V04.bi","V05.bi","V06.bi","V07.bi","V08.bi","V09.bi","V10.bi","V11.bi","V12.bi","V13.bi","V14.bi","V15.bi","V16.bi","V17.bi","V18.bi","V19.bi","V20.bi","NVarbs.bi","Turn.Duration.C","CharPerWord.C","WordPerSec.C")

full.names<-data.frame(fullname, variable)


df.res2<-merge(df.res2, full.names, by="variable")

df.res2$hilo<-"lo"
df.res2$hilo[df.res2$value >=abs(min(df.res2$value))]<-'hi'
df.res2$hilo<-factor(df.res2$hilo, levels = c("lo", "hi"))
df.line<-abs(min(df.res2$value))

ggbarplot(df.res2, 
                x = "fullname", 
                y = "value",
                sort.val = "asc",
                add = "segments",
                rotate = TRUE, 
                color = "hilo",
                fill = "hilo",
                palette = "startrek",
                xlab = FALSE,
                ylab = FALSE, 
                legend = "none")
```

```
df2$Prediction<-predict(df.rf, df2)
confusionMatrix(df2$Prediction, df2$NFP.bi)
df.rf.predictions<-predict(df.rf, type="prob")
df.rf.pred<-prediction(df.rf.predictions[,2], df$NFP.bi)
performance(df.rf.pred, measure="auc")@y.values

df.rfscr<-rfsrc(NFP.bi~.,data=df2, ntree=500, mtry=best.m, importance = TRUE)
find.interaction(df.rfscr, method="vimp")
```

```
glmer.FP.with <-glmer(NFP.bi~Turn.Duration.C+ CharPerWord.C+ WordPerSec.C+(1|Speaker_Number), data=df[df$NVarbs.bi=="1",], family="binomial", control = glmerControl(optCtrl =list(maxfun = 2e4), optimizer = "bobyqa"))
glmer.FP.without<-glmer(NFP.bi~Turn.Duration.C+ CharPerWord.C+ WordPerSec.C+(1|Speaker_Number), data=df[df$NVarbs.bi=="0",], family="binomial", control = glmerControl(optCtrl =list(maxfun = 2e4), optimizer = "bobyqa"))

effects.Duration.with<- effects::effect(term="Turn.Duration.C", mod=glmer.FP.with)
effects.Duration.without<- effects::effect(term="Turn.Duration.C", mod=glmer.FP.without)

ggplot()+
geom_count(data=df, aes(x=Turn.Duration.C,y=as.numeric(as.character(NFP.bi)), color=NVarbs.bi), alpha=0.3, show.legend = F) +
  scale_color_manual(values=c("#5C88DAFF","#CC0C00FF" ))+
  geom_point(data=as.data.frame(effects.Duration.with), aes(x=Turn.Duration.C, y=fit), color="#5C88DAFF")+
  geom_line(data=as.data.frame(effects.Duration.with), aes(x=Turn.Duration.C, y=fit), color="#5C88DAFF")+
  geom_ribbon(data=as.data.frame(effects.Duration.with), aes(x=Turn.Duration.C, ymin=lower, ymax=upper), alpha= 0.3, fill="#5C88DAFF")+
  geom_point(data=as.data.frame(effects.Duration.without), aes(x=Turn.Duration.C, y=fit), color="#CC0C00FF")+
  geom_line(data=as.data.frame(effects.Duration.without), aes(x=Turn.Duration.C, y=fit), color="#CC0C00FF")+
  geom_ribbon(data=as.data.frame(effects.Duration.without), aes(x=Turn.Duration.C, ymin=lower, ymax=upper), alpha= 0.3, fill="#CC0C00FF")+
  labs(x="Turn Duration (centered & scaled)", y="Filled Pauses")+
  theme_classic2()+
  scale_y_continuous(breaks=c(0,1))+
  annotate("text",label="With Variable Contexts", color = "#5C88DAFF" , x=1, y=.25, hjust=0)+
  annotate("text",label="Without Variable Contexts",color="#CC0C00FF", x= 1, y=.75, hjust=1)
```

```
lmer.SPW<-lmer(SilPerWord.log~NVarbs.bi+Turn.Duration.C+ CharPerWord.C+ WordPerSec.C+(1|Speaker_Number), data=df, REML=FALSE )

summary(lmer.SPW)
```

```
## Linear mixed model fit by maximum likelihood . t-tests use Satterthwaite's
##   method [lmerModLmerTest]
## Formula: SilPerWord.log ~ NVarbs.bi + Turn.Duration.C + CharPerWord.C +  
##     WordPerSec.C + (1 | Speaker_Number)
##    Data: df
## 
##      AIC      BIC   logLik deviance df.resid 
##  13425.9  13474.1  -6706.0  13411.9     7154 
## 
## Scaled residuals: 
##     Min      1Q  Median      3Q     Max 
## -9.2921 -0.4537  0.1327  0.5830  4.4744 
## 
## Random effects:
##  Groups         Name        Variance Std.Dev.
##  Speaker_Number (Intercept) 0.1179   0.3434  
##  Residual                   0.3739   0.6115  
## Number of obs: 7161, groups:  Speaker_Number, 34
## 
## Fixed effects:
##                   Estimate Std. Error         df t value Pr(>|t|)    
## (Intercept)     -2.431e+00  6.018e-02  3.498e+01 -40.401  < 2e-16 ***
## NVarbs.bi1      -1.100e-02  1.634e-02  7.135e+03  -0.673    0.501    
## Turn.Duration.C -3.408e-02  8.230e-03  7.143e+03  -4.142 3.49e-05 ***
## CharPerWord.C   -6.570e-02  7.640e-03  7.133e+03  -8.599  < 2e-16 ***
## WordPerSec.C    -3.821e-01  8.337e-03  7.149e+03 -45.825  < 2e-16 ***
## ---
## Signif. codes:  0 '***' 0.001 '**' 0.01 '*' 0.05 '.' 0.1 ' ' 1
## 
## Correlation of Fixed Effects:
##             (Intr) NVrb.1 Tr.D.C ChPW.C
## NVarbs.bi1  -0.129                     
## Turn.Drtn.C  0.044 -0.344              
## CharPrWrd.C -0.001  0.003 -0.147       
## WordPerSc.C  0.032 -0.228 -0.120  0.272
```

```
r.squaredGLMM(lmer.SPW)
```

```
## Warning: 'r.squaredGLMM' now calculates a revised statistic. See the help page.
```

```
##            R2m       R2c
## [1,] 0.2297295 0.4144013
```

```
vif(lmer.SPW)
```

```
##       NVarbs.bi Turn.Duration.C   CharPerWord.C    WordPerSec.C 
##        1.238328        1.202946        1.096510        1.184069
```

```
detach("package:lmerTest", unload=TRUE)
collin.fnc.mer(lmer(SilPerWord.log~NVarbs.bi+Turn.Duration.C+ CharPerWord.C+ WordPerSec.C+(1|Speaker_Number), data=df, REML=FALSE ))$cnumber
```

```
## [1] 2.746263
```

```
library(lmerTest)
```

```
## 
## Attaching package: 'lmerTest'
```

```
## The following object is masked from 'package:lme4':
## 
##     lmer
```

```
## The following object is masked from 'package:stats':
## 
##     step
```

```
lmer.SPW.all<-lmer(SilPerWord.log~V01.bi+V02.bi+V03.bi+V04.bi+V05.bi+V06.bi+V07.bi+V08.bi+V09.bi+V10.bi+V11.bi+V12.bi+V13.bi+V14.bi+V15.bi+V16.bi+V17.bi+V18.bi+V19.bi+V20.bi+Turn.Duration.C+ CharPerWord.C+ WordPerSec.C+(1|Speaker_Number), data=df, REML=FALSE)

summary(lmer.SPW.all)
```

```
## Linear mixed model fit by maximum likelihood . t-tests use Satterthwaite's
##   method [lmerModLmerTest]
## Formula: SilPerWord.log ~ V01.bi + V02.bi + V03.bi + V04.bi + V05.bi +  
##     V06.bi + V07.bi + V08.bi + V09.bi + V10.bi + V11.bi + V12.bi +  
##     V13.bi + V14.bi + V15.bi + V16.bi + V17.bi + V18.bi + V19.bi +  
##     V20.bi + Turn.Duration.C + CharPerWord.C + WordPerSec.C +  
##     (1 | Speaker_Number)
##    Data: df
## 
##      AIC      BIC   logLik deviance df.resid 
##  13402.8  13581.6  -6675.4  13350.8     7135 
## 
## Scaled residuals: 
##     Min      1Q  Median      3Q     Max 
## -9.3288 -0.4459  0.1335  0.5765  4.3952 
## 
## Random effects:
##  Groups         Name        Variance Std.Dev.
##  Speaker_Number (Intercept) 0.1168   0.3418  
##  Residual                   0.3707   0.6089  
## Number of obs: 7161, groups:  Speaker_Number, 34
## 
## Fixed effects:
##                   Estimate Std. Error         df t value Pr(>|t|)    
## (Intercept)     -2.434e+00  5.977e-02  3.466e+01 -40.718  < 2e-16 ***
## V01.bi1         -2.869e-02  4.278e-02  7.129e+03  -0.671 0.502412    
## V02.bi1          6.947e-02  1.056e-01  7.129e+03   0.658 0.510670    
## V03.bi1         -1.811e-02  1.990e-02  7.131e+03  -0.910 0.363048    
## V04.bi1         -1.690e-01  1.161e-01  7.130e+03  -1.455 0.145628    
## V05.bi1          2.458e-02  2.281e-02  7.132e+03   1.078 0.281255    
## V06.bi1         -6.514e-02  9.082e-02  7.130e+03  -0.717 0.473217    
## V07.bi1         -4.756e-01  1.410e-01  7.128e+03  -3.373 0.000747 ***
## V08.bi1          5.601e-02  2.960e-02  7.130e+03   1.892 0.058509 .  
## V09.bi1          1.544e-02  3.665e-02  7.130e+03   0.421 0.673463    
## V10.bi1         -2.276e-03  2.948e-02  7.133e+03  -0.077 0.938479    
## V11.bi1          6.591e-02  4.250e-02  7.133e+03   1.551 0.121009    
## V12.bi1         -1.096e-01  4.595e-02  7.129e+03  -2.386 0.017074 *  
## V13.bi1         -5.980e-03  3.027e-02  7.129e+03  -0.198 0.843404    
## V14.bi1         -9.173e-02  4.234e-02  7.130e+03  -2.166 0.030306 *  
## V15.bi1         -1.988e-01  9.786e-02  7.130e+03  -2.031 0.042254 *  
## V16.bi1         -2.268e-01  6.073e-02  7.131e+03  -3.735 0.000190 ***
## V17.bi1         -1.575e-02  9.265e-02  7.129e+03  -0.170 0.865032    
## V18.bi1          5.942e-03  1.641e-01  7.128e+03   0.036 0.971114    
## V19.bi1          1.200e-01  8.830e-02  7.130e+03   1.359 0.174074    
## V20.bi1          1.278e-01  4.681e-02  7.133e+03   2.730 0.006352 ** 
## Turn.Duration.C -3.440e-02  8.507e-03  7.144e+03  -4.044 5.31e-05 ***
## CharPerWord.C   -6.498e-02  7.675e-03  7.133e+03  -8.466  < 2e-16 ***
## WordPerSec.C    -3.820e-01  8.363e-03  7.148e+03 -45.677  < 2e-16 ***
## ---
## Signif. codes:  0 '***' 0.001 '**' 0.01 '*' 0.05 '.' 0.1 ' ' 1
```

```
## 
## Correlation matrix not shown by default, as p = 24 > 12.
## Use print(x, correlation=TRUE)  or
##     vcov(x)        if you need it
```

```
r.squaredGLMM(lmer.SPW.all)
```

```
##            R2m       R2c
## [1,] 0.2348764 0.4182276
```

```
vif(lmer.SPW.all)
```

```
##          V01.bi          V02.bi          V03.bi          V04.bi          V05.bi 
##        1.029990        1.010161        1.111690        1.006090        1.070023 
##          V06.bi          V07.bi          V08.bi          V09.bi          V10.bi 
##        1.007452        1.009090        1.056404        1.028001        1.036464 
##          V11.bi          V12.bi          V13.bi          V14.bi          V15.bi 
##        1.034690        1.018771        1.029763        1.026138        1.018918 
##          V16.bi          V17.bi          V18.bi          V19.bi          V20.bi 
##        1.023684        1.008687        1.010363        1.014456        1.038928 
## Turn.Duration.C   CharPerWord.C    WordPerSec.C 
##        1.296302        1.116109        1.201674
```

```
detach("package:lmerTest", unload=TRUE)
collin.fnc.mer(lmer(SilPerWord.log~V01.bi+V02.bi+V03.bi+V04.bi+V05.bi+V06.bi+V07.bi+V08.bi+V09.bi+V10.bi+V11.bi+V12.bi+V13.bi+V14.bi+V15.bi+V16.bi+V17.bi+V18.bi+V19.bi+V20.bi+Turn.Duration.C+ CharPerWord.C+ WordPerSec.C+(1|Speaker_Number), data=df, REML=FALSE))$cnumber
```

```
## [1] 2.783462
```

```
df2<-df%>%
dplyr:: select(SilPerWord.log,V01.bi,V02.bi,V03.bi,V04.bi,V05.bi,V06.bi,V07.bi,V08.bi,V09.bi,V10.bi,V11.bi,V12.bi,V13.bi,V14.bi,V15.bi,V16.bi,V17.bi,V18.bi,V19.bi,V20.bi,NVarbs.bi,Turn.Duration.C,CharPerWord.C,WordPerSec.C)

set.seed(2000)
mtry<-tuneRF(df2[-1], df2$SilPerWord.log, ntreeTry=128, stepFactor=1.35, improve=0.01, trace=FALSE, plot=FALSE)

best.m<-min(mtry[mtry[,2] == min(mtry[,2]),1])

df.rf<-randomForest(SilPerWord.log~.,data=df2, ntree=500, mtry=best.m, replace=FALSE, keep.forest=TRUE, keep.inbag=TRUE, importance=TRUE, proximity=TRUE)

df.res3<-permimp(df.rf, conditional=TRUE, progressBar=FALSE, do_check=FALSE)

df.res4<-gather(data.frame(as.list(df.res3$values)), variable, value)


df.res4<-merge(df.res4, full.names, by="variable")

df.res4$hilo<-"lo"
df.res4$hilo[df.res4$value >=abs(min(df.res4$value))]<-'hi'
df.res4$hilo<-factor(df.res4$hilo, levels = c("lo", "hi"))
df.line<-abs(min(df.res4$value))

ggbarplot(df.res4, 
                x = "fullname", 
                y = "value",
                sort.val = "asc",
                add = "segments",
                rotate = TRUE, 
                color = "hilo",
                fill = "hilo",
                palette = "startrek",
                xlab = FALSE,
                ylab = FALSE, 
                legend = "none")
```

```
df.rf.predictions<-predict(df.rf)
RMSE<-sqrt(sum((df.rf.predictions - df2$SilPerWord.log)^2/length(df.rf.predictions)))
print(RMSE)
```

```
bar1<- ggbarplot(df.res2, 
                x = "fullname", 
                y = "value",
                sort.val = "asc",
                add = "segments",
                rotate = TRUE, 
                color = "hilo",
                fill = "hilo",
                palette = "startrek",
                xlab = FALSE,
                ylab = FALSE, 
                legend = "none")

ggsave("~/Box Sync/Complexity Projects/Complexity and Choice/Manuscripts/PlosOne/ThirdSubmission/Figures/Figure4.png",bar1, width=6, height=6, units="in", dpi = 1000, type = "cairo")


bar2<- ggbarplot(df.res4, 
                x = "fullname", 
                y = "value",
                sort.val = "asc",
                add = "segments",
                rotate = TRUE, 
                color = "hilo",
                fill = "hilo",
                palette = "startrek",
                xlab = FALSE,
                ylab = FALSE, 
                legend = "none")

ggsave("~/Box Sync/Complexity Projects/Complexity and Choice/Manuscripts/PlosOne/ThirdSubmission/Figures/Figure6.png",bar2, width=6, height=6, units="in", dpi = 1000, type = "cairo")


gg<- ggplot()+
geom_count(data=df, aes(x=Turn.Duration.C,y=as.numeric(as.character(NFP.bi)), color=NVarbs.bi), alpha=0.3, show.legend = F) +
  scale_color_manual(values=c("#5C88DAFF","#CC0C00FF" ))+
  geom_point(data=as.data.frame(effects.Duration.with), aes(x=Turn.Duration.C, y=fit), color="#5C88DAFF")+
  geom_line(data=as.data.frame(effects.Duration.with), aes(x=Turn.Duration.C, y=fit), color="#5C88DAFF")+
  geom_ribbon(data=as.data.frame(effects.Duration.with), aes(x=Turn.Duration.C, ymin=lower, ymax=upper), alpha= 0.3, fill="#5C88DAFF")+
  geom_point(data=as.data.frame(effects.Duration.without), aes(x=Turn.Duration.C, y=fit), color="#CC0C00FF")+
  geom_line(data=as.data.frame(effects.Duration.without), aes(x=Turn.Duration.C, y=fit), color="#CC0C00FF")+
  geom_ribbon(data=as.data.frame(effects.Duration.without), aes(x=Turn.Duration.C, ymin=lower, ymax=upper), alpha= 0.3, fill="#CC0C00FF")+
  labs(x="Turn Duration (centered & scaled)", y="Filled Pauses")+
  theme_classic2()+
  scale_y_continuous(breaks=c(0,1))+
  annotate("text",label="With Variable Contexts", color = "#5C88DAFF" , x=1, y=.25, hjust=0)+
  annotate("text",label="Without Variable Contexts",color="#CC0C00FF", x= 1, y=.75, hjust=1)

ggsave("~/Box Sync/Complexity Projects/Complexity and Choice/Manuscripts/PlosOne/ThirdSubmission/Figures/Figure5.png",gg, width=5, height=5, units="in", dpi = 1000, type = "cairo")
```
